# Supplementary material for: Nanoscale Dynamism of Actin Enables Secretory Function in Cytolytic Cells
Source: Curr Biol. 2018 Feb 19;28(4):489–502.e9. doi: 10.1016/j.cub.2017.12.044 (PMC5835143; doi:10.1016/j.cub.2017.12.044)
Supplement: Document S1. Figures S1–S5 [file mmc1.pdf]

Current Biology, Volume *28*

Supplemental Information

Nanoscale Dynamism of Actin Enables Secretory Function in Cytolytic Cells

Alexandre F. Carisey, Emily M. Mace, Mezida B. Saeed, Daniel M. Davis, and Jordan S. Orange

Figure S1

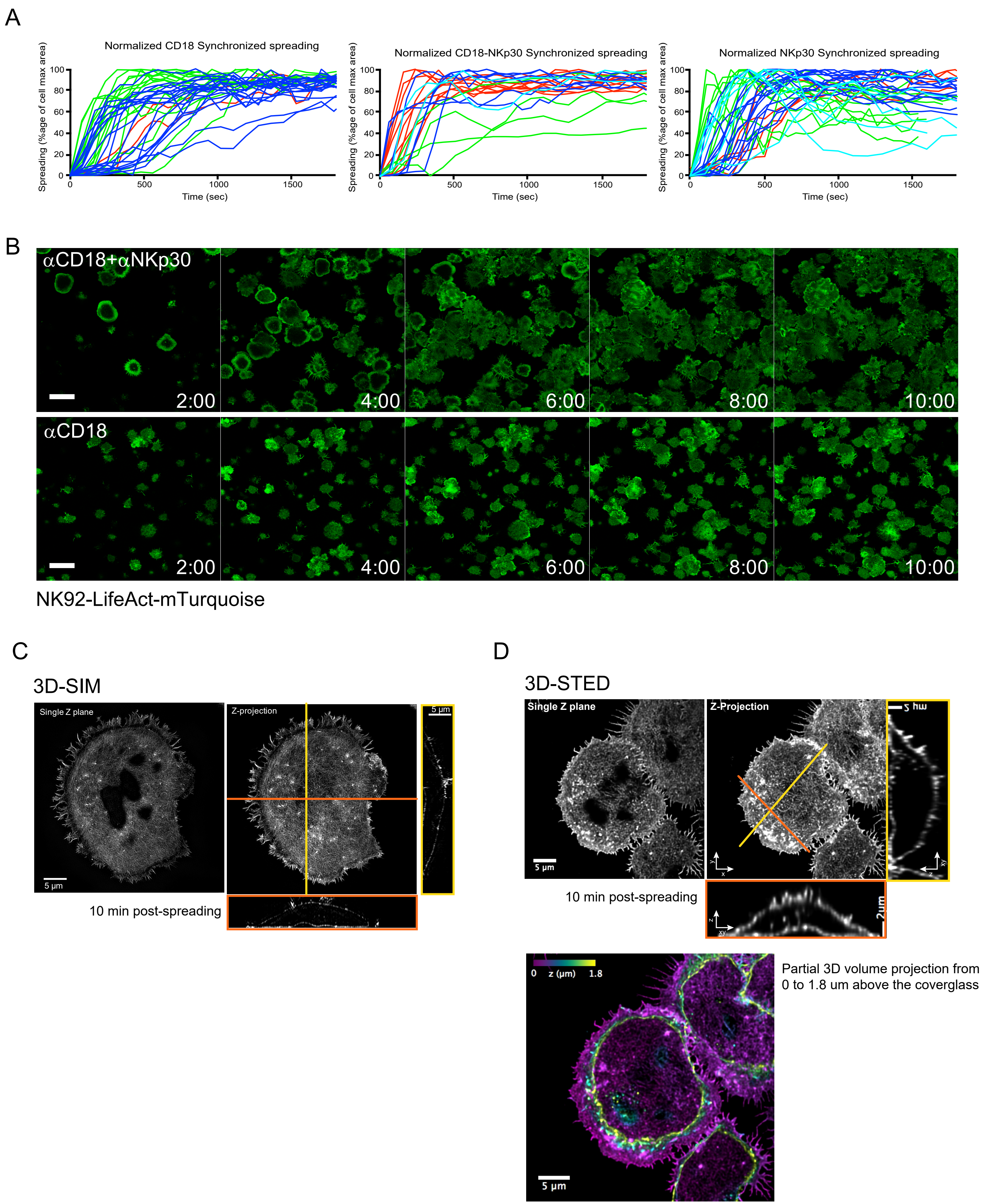
A
Normalized CD18 Synchronized spreading
Normalized CD18-NKp30 Synchronized spreading
Normalized NKp30 Synchronized spreading
Spreading (%age of cell max area)
Time (sec)
B
αCD18+αNKp30
αCD18
2:00
4:00
6:00
8:00
10:00
NK92-LifeAct-mTurquoise
C
3D-SIM
Single Z plane
Z-projection
5 µm
10 min post-spreading
D
3D-STED
Single Z plane
Z-Projection
5 µm
2µm
10 min post-spreading
0 z (µm) 1.8
Partial 3D volume projection from 0 to 1.8 um above the coverglass

Figure S1. Mature NK cell lytic synapse is defined by a pervasive actin network. Related to Figure 1.

(A) Individual spreading measurements traces for NK92.LifeAct-mEmerald cells plated on the indicated substrate and imaged by confocal microscopy at the plane of the glass. Each track has been aligned in time to the beginning of each cell contact with the glass and normalized to the maximum cell area achieved. N = 37, 21 and 35 cells respectively per condition from 3, 4 and 4 independent experiments. **(B)** Representative frames (from 7 independent repeats) from time lapse imaging of larger fields of view of NK92.LifeAct-mEmerald cells plated on the indicated substrate, imaged by confocal microscopy at the plane of the glass showing the consistency of the detection of a bright circular lamellipodium in presence of anti-NKp30 antibody. Scale bar = 20 µm. **(C)** 3D-SIM reconstruction of NK92 cell stained for F-actin by phalloidin 488 and its orthogonal projection illustrating the irregularity of the plasma membrane of activated NK cells. Scale bar = 5 µm. **(D)** Similar sample as **(C)** imaged by 3D-STED microscopy. Scale bar = 5 µm.

Figure S2

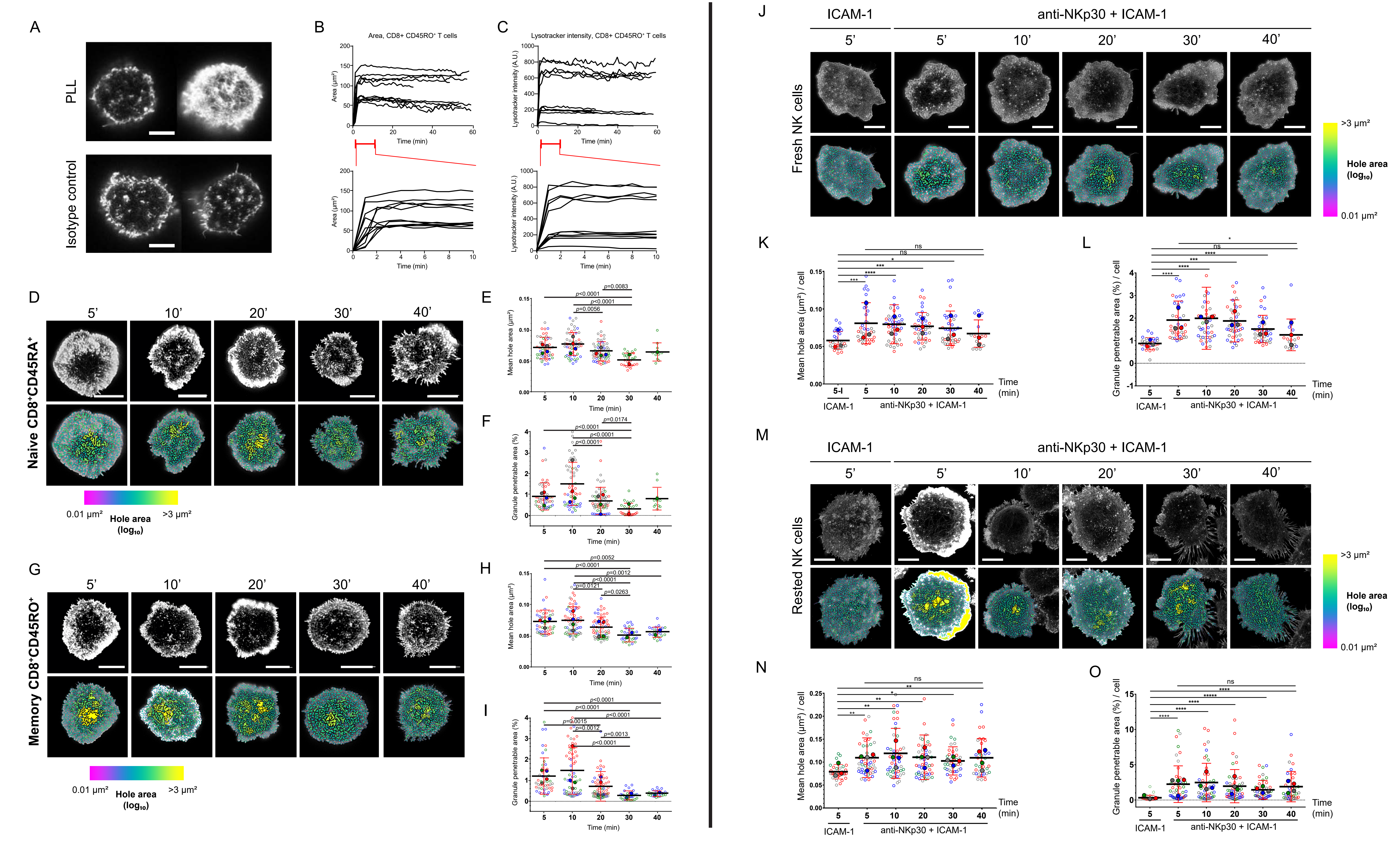

A
PLL
Isotype control
B
Area, CD8+ CD45RO+ T cells
Area (µm²)
Time (min)
C
Lysotracker intensity, CD8+ CD45RO+ T cells
Lysotracker intensity (A.U.)
Time (min)
D
5’
10’
20’
30’
40’
Naive CD8+CD45RA+
0.01 µm²
Hole area (log10)
>3 µm²
E
Mean hole area (µm²)
p<0.0001
p=0.0083
p<0.0001
p=0.0056
Time (min)
F
Granule penetrable area (%)
p<0.0001
p=0.0174
p<0.0001
p<0.0001
Time (min)
G
5’
10’
20’
30’
40’
Memory CD8+CD45RO+
0.01 µm²
Hole area (log10)
>3 µm²
H
Mean hole area (µm²)
p=0.0052
p<0.0001
p=0.0012
p<0.0001
p=0.0121
p=0.0263
Time (min)
I
Granule penetrable area (%)
p<0.0001
p<0.0001
p<0.0001
p=0.0015
p=0.0012
p=0.0013
p<0.0001
Time (min)
J
ICAM-1
anti-NKp30 + ICAM-1
5’
5’
10’
20’
30’
40’
Fresh NK cells
>3 µm²
Hole area (log10)
0.01 µm²
K
Mean hole area (µm²) / cell
ns
ns
*
***
****
***
5-I
5
10
20
30
40
Time (min)
ICAM-1
anti-NKp30 + ICAM-1
L
Granule penetrable area (%) / cell
*
ns
****
***
****
****
Time (min)
ICAM-1
anti-NKp30 + ICAM-1
M
ICAM-1
anti-NKp30 + ICAM-1
5’
5’
10’
20’
30’
40’
Rested NK cells
>3 µm²
Hole area (log10)
0.01 µm²
N
Mean hole area (µm²) / cell
ns
**
*
**
**
**
Time (min)
ICAM-1
anti-NKp30 + ICAM-1
O
Granule penetrable area (%) / cell
ns
****
*****
****
****
****
Time (min)
ICAM-1
anti-NKp30 + ICAM-1

Figure S2. Granule-permissive sized clearances persist following degranulation and are a feature of primary cytotoxic T cells and NK cells. Related to Figure 2.

(A) Representative images (selected from 17 cells) of primary T cells plated on PLL or isotype control coated glass for 20 minutes and stained for F-actin, illustrating the dense cortical actin mesh observed in the absence of activation signal. Scale bar = 5 µm. **(B)** Individual spreading measurements traces for primary $CD8^+$ $CD45RO^+$ cells loaded with LysoTracker Red and plated on anti-CD3 and anti-CD28 and imaged by confocal microscopy at the plane of the glass. Each track has been aligned in time to the beginning of each cell contact with the glass. Below, an inset shows the first 10 minutes of the time lapse. **(C)** Measurement of LysoTracker fluorescence intensity at the plane of the synapse during the time lapse imaging experiment described in **(B)**. N = 11 cells pooled from 2 independent repeats in (B) and **(C)**. Primary human naïve [$CD8^+$ $CD45RA^+$] **(D)** or memory [$CD8^+$ $CD45RO^+$] **(G)** T cells isolated from peripheral blood of healthy donors and activated on anti-CD3 and anti-CD28 for times indicated prior to fixation and staining with Phalloidin AlexaFluor 488. Cells were imaged by time-gated STED microscopy. Scale bar = 5 µm. Mean hole area per cell was plotted on the images (lower panel, heat map) and is shown in graphs on right **(E, H)** along the percentage of the granule penetrable area per cell **(F, I)**. Images shown are representative of those from 4 healthy donors which were pooled for quantitative analysis. N = 10 cells per donor, each donor colored individually in graphs. *p* value calculated using one-way ANOVA Kruskal-Wallis test (Dunn's). Primary human fresh **(J)** or rested **(M)** NK cells isolated from peripheral blood of healthy donors and activated on ICAM-1 or a combination of ICAM-1 and anti-NKp30 for times indicated prior to fixation and staining with Phalloidin AlexaFluor 488. Cells were imaged by time-gated STED microscopy. Scale bar = 5 µm. Mean hole area per cell was plotted back on the images (lower panel, heat map) and is shown in graphs directly below **(K, N)** along the percentage of the granule penetrable area per cell **(L, O)**. Images shown are representative of those from 3 (fresh NK cells) or 4 (rested NK cells) healthy donors which were pooled for quantitative

analysis. N = 10 cells per donor, each donor is colored individually in graphs. *p* value calculated using one-way ANOVA Kruskal-Wallis test (Dunn’s).

Figure S3

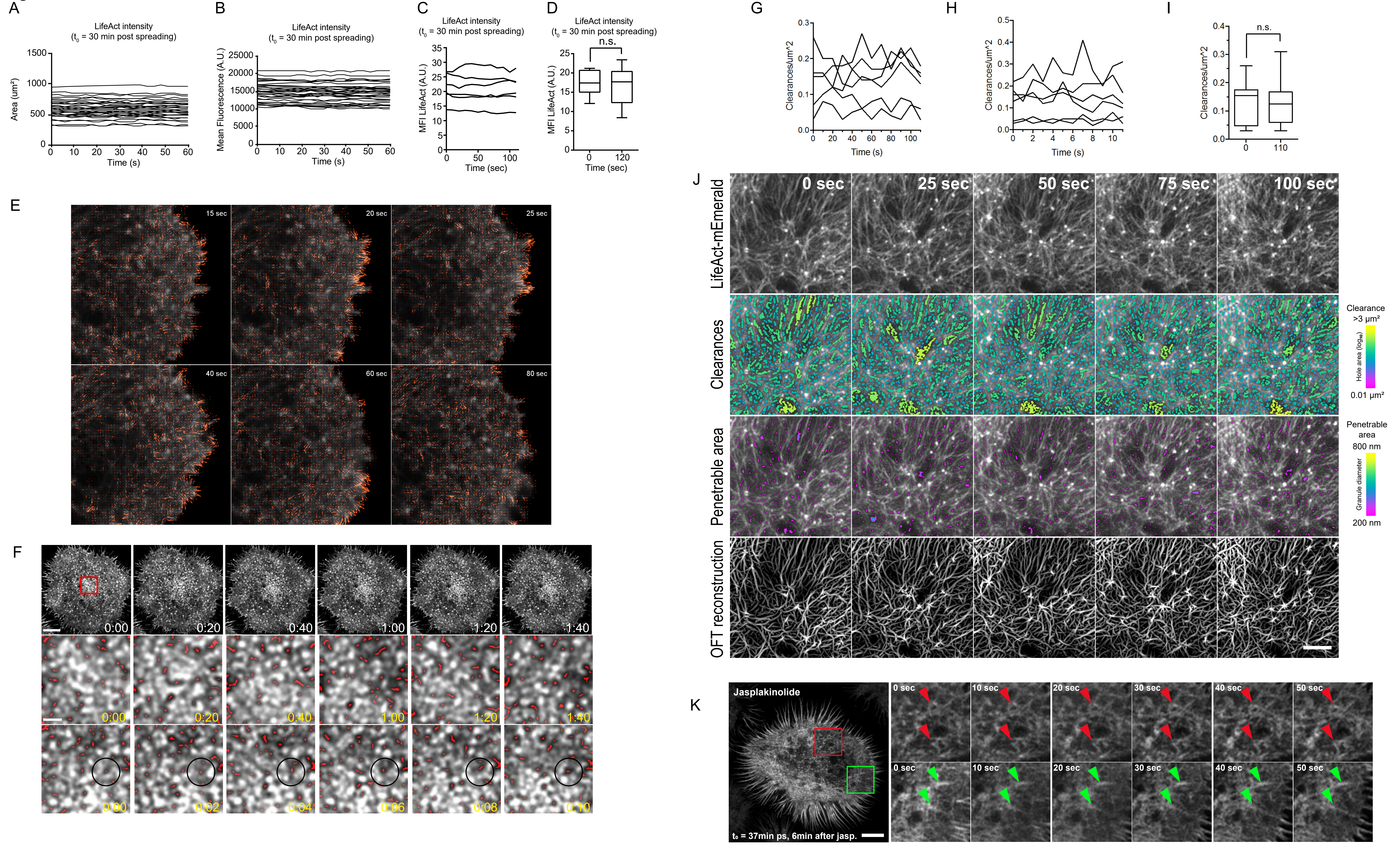
A
LifeAct intensity
(t_0 = 30 min post spreading)
Area (um²)
Time (s)
B
LifeAct intensity
(t_0 = 30 min post spreading)
Mean Fluorescence (A.U.)
Time (s)
C
LifeAct intensity
(t_0 = 30 min post spreading)
MFI LifeAct (A.U.)
Time (sec)
D
LifeAct intensity
(t_0 = 30 min post spreading)
n.s.
MFI LifeAct (A.U.)
Time (sec)
G
Clearances/um^2
Time (s)
H
Clearances/um^2
Time (s)
I
n.s.
Clearances/um^2
E
15 sec
20 sec
25 sec
40 sec
60 sec
80 sec
F
0:00
0:20
0:40
1:00
1:20
1:40
0:00
0:02
0:04
0:06
0:08
0:10
J
0 sec
25 sec
50 sec
75 sec
100 sec
LifeAct-mEmerald
Clearances
Penetrable area
OFT reconstruction
Clearance
>3 µm²
Hole area (log10)
0.01 µm²
Penetrable area
800 nm
Granule diameter
200 nm
K
Jasplakinolide
t₀ = 37min ps, 6min after jasp.
0 sec
10 sec
20 sec
30 sec
40 sec
50 sec

Figure S3. Global F-actin stability at the mature NK IS is coupled to local actin dynamism. Related to Figure 3.

NK92 LifeAct.mTurquoise cells were activated for 30 minutes prior to initiation of imaging by TIRF-SIM or time gated STED and reached a stable footprint size **(A)**. Filamentous actin was detected using LifeAct-mEmerald and mean fluorescence intensity over time throughout the time of imaging was measured in TIRF-SIM **(B)** or time gated STED **(C)**. **(D)** The MFI of LifeAct did not change significantly over the time course of imaging as calculated from 7 cells from 1 experiment (representative of 3 experiments). *p* value calculated by Mann-Whitney unpaired two-tailed test. **(E)** Frames from the optical flow analysis of the LifeAct-mEmerald fluorescent signal at the edge of NK92 cell following 30 minutes of activation on antibody-coated glass. Optical flow is calculated using Farnback's algorithm for every frame (5 seconds) but displayed at critical timepoints illustrating the concomitance of actin flow with membrane edge movement (see Movie S3). **(F)** Time lapse series of a single cell representative of 25 cells from 3 independent experiments imaged by live time-gated STED microscopy. Insets below are from the same cell but captured at different frame rate (1 frame every 10 seconds or 1 frame per second) to illustrate the fast and continuous dynamism. Scale bar = 5 µm (top panel), 0.5 µm (bottom panel). **(G, H)** Measurement of the clearances/μm^2 over time confirms the dynamism of the clearances observed in **(F)** using both slow (1 frame every 10 seconds, 5 cells, **G**) and fast datasets (1 frame every second, 6 cells, **H**). **(I)** The overall distribution of the number of clearances did not change significantly over the time course of imaging (110 seconds, 1 frame per second) as calculated from 10 cells from 1 experiment (representative of 3 experiments). *p* value calculated by Mann-Whitney unpaired two-tailed test. **(J)** Larger field of view from the dataset presented in Figure 3D and in Movie S4. Below the fluorescent LifeAct-mEmerald reporter signal, the clearances have been plotted onto the original dataset. The third row shows the clearances compatible with the extrusion of a granule with a diameter ranging from 200 to 800nm. The last row displays the OFT reconstruction of the same area emphasizing the local dynamism of the actin fibers. Scale bar = 1 µm. **(K)** Stabilization of the F-actin

cytoskeleton with 1 μM jasplakinolide administered 30 minutes after activation (see movie S5). Arrowheads highlight stable F-actin structures. Representative cell from 28 cells from 3 independent repeats.

Figure S4

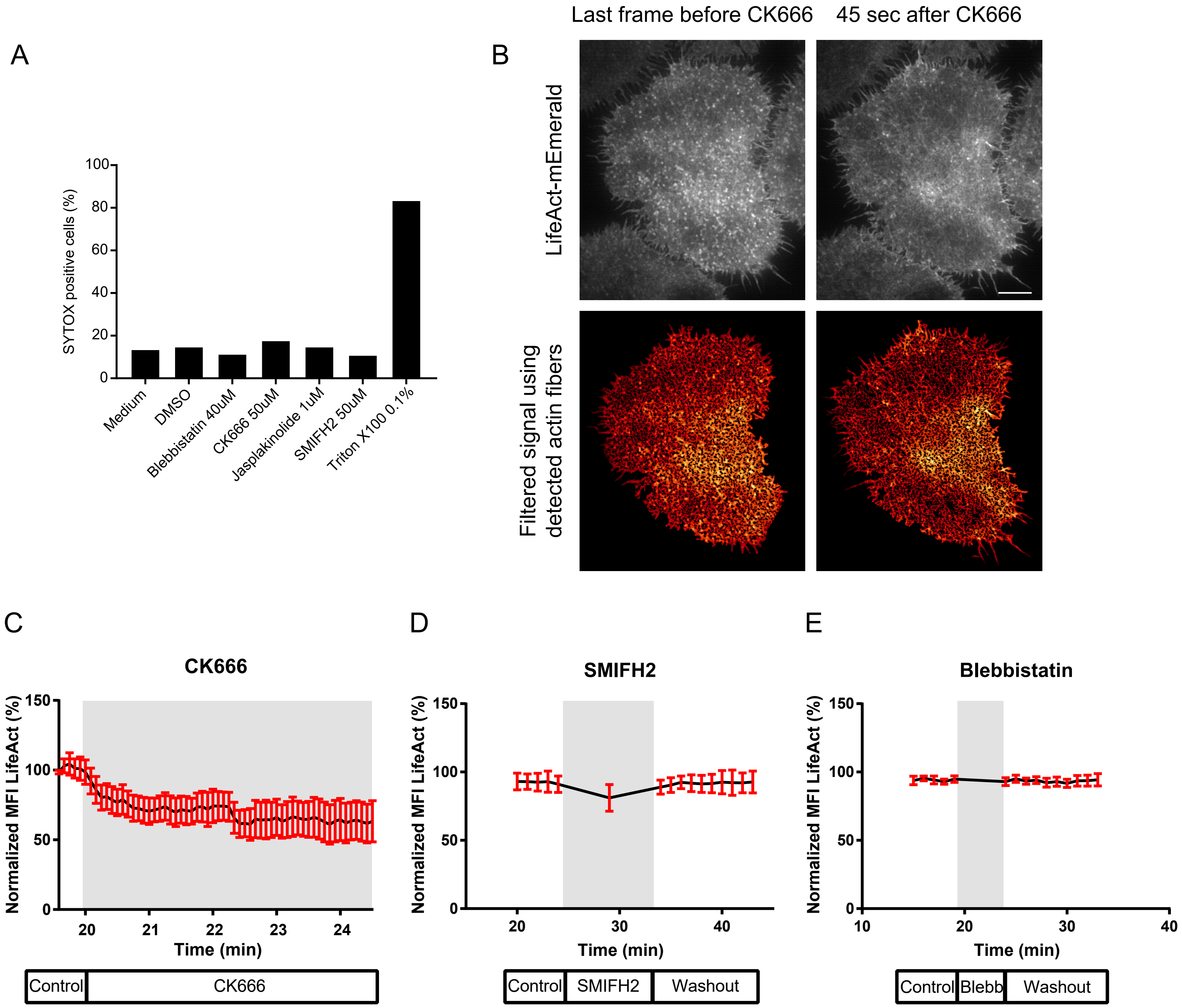
A
SYTOX positive cells (%)
100
80
60
40
20
0
Medium
DMSO
Blebbistatin 40uM
CK666 50uM
Jasplakinolide 1uM
SMIFH2 50uM
Triton X100 0.1%
B
Last frame before CK666
45 sec after CK666
LifeAct-mEmerald
Filtered signal using detected actin fibers
C
CK666
Normalized MFI LifeAct (%)
150
100
50
0
20
21
22
23
24
Time (min)
Control
CK666
D
SMIFH2
Normalized MFI LifeAct (%)
150
100
50
0
20
30
40
Time (min)
Control
SMIFH2
Washout
E
Blebbistatin
Normalized MFI LifeAct (%)
150
100
50
0
10
20
30
40
Time (min)
Control
Blebb
Washout

Figure S4. The mature immune synapse is characterized by intense Arp2/3 based actin remodeling. Related to Figure 4.

(A) Viability of NK92.LifeAct-mEmerald cells tested after treatment with the indicating drug for 10 minutes following 20 minutes of activation on antibody coated glass. SYTOX was added while washing out the drug and imaged by confocal microscopy. The proportion of SYTOX positive cells indicates cell death (Triton X100 is a positive control). None of the drug treatment used in this study is significantly more toxic to NK92 cells than the control (medium) or vehicle control (DMSO). **(B)** Detection of the actin filaments from the TIRF-SIM dataset used in Figure 4E illustrating the increased sized of the clearances and disappearance of the actin foci following the addition of 50 µM of CK666 to activated NK92 cells on antibody coated glass (see Movie S7). Scale bar = 5 µm. **(C)** Normalized mean fluorescence intensity of the LifeAct-mEmerald signal at the immune synapse of NK92 cells activated for 20 minutes on antibody coated glass and treated with 50 µM of CK666. Cells are imaged every 5 seconds and the intensity is normalized to 100% for each cell 5 seconds before the addition of the inhibitor. Data from 10 cells, representative from 6 repeats. Error bars indicate 95% CI. **(D)** Normalized mean fluorescence intensity of the LifeAct-mEmerald signal at the immune synapse of NK92 cells activated for 20 minutes on antibody coated glass and treated with 50 µM of SMIFH2. Cells are imaged every minute and the intensity is normalized to 100% for each cell 5 minutes before the addition of the inhibitor. One time point is recorded during the treatment and then for 10 minutes, every minute, after drug washout. No significant change in the total actin mass at the IS is observed during or after the drug treatment. Data from 10 cells, representative from 6 repeats. Error bars indicate 95% CI. **(E)** Normalized mean fluorescence intensity of the LifeAct-mEmerald signal at the immune synapse of NK92 cells activated for 20 minutes on antibody coated glass and treated with 40 µM of blebbistatin. Cells are imaged every minute and the intensity is normalized to 100% for each cell 5 minutes before the addition of the inhibitor. Then, cells are imaged every minute for 10 minutes after drug washout. No

significant change in the total actin mass at the IS is observed after the drug treatment. Data from 10 cells, representative from 6 repeats. Error bars indicate 95% CI.

Figure S5

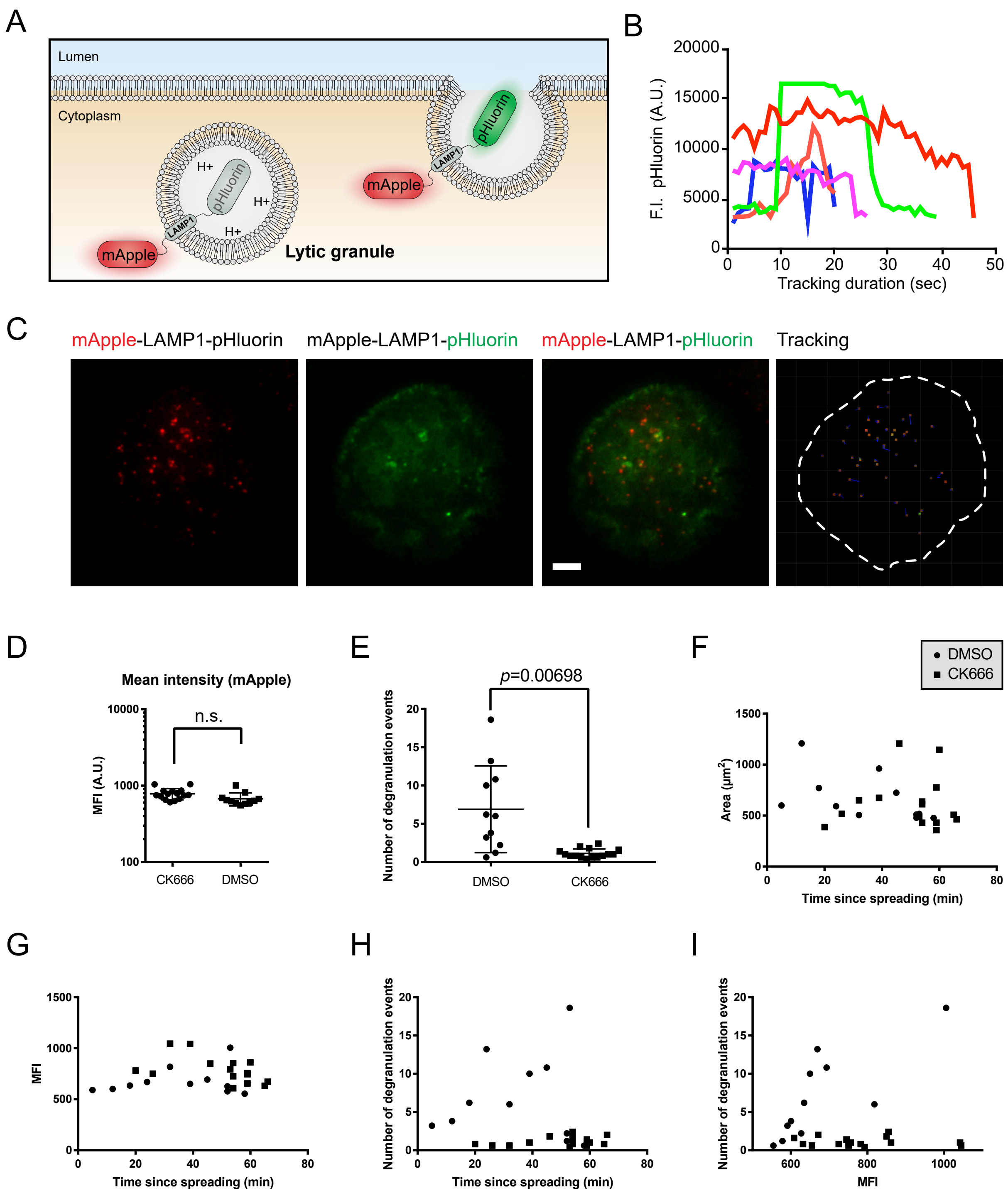

A
Lumen
Cytoplasm
pHluorin
LAMP1
mApple
H+
Lytic granule
B
F.I. pHluorin (A.U.)
Tracking duration (sec)
C
mApple-LAMP1-pHluorin
Tracking
D
Mean intensity (mApple)
n.s.
MFI (A.U.)
CK666
DMSO
E
p=0.00698
Number of degranulation events
DMSO
CK666
F
DMSO
CK666
Area (μm²)
Time since spreading (min)
G
MFI
Time since spreading (min)
H
Number of degranulation events
Time since spreading (min)
I
Number of degranulation events
MFI

Figure S5. Analysis of degranulation using mApple-LAMP1-pHluorin expressing NK92. Related to Figure 5.

(A) Cartoon illustrating the mode of action of the mApple-LAMP1-pHluorin degranulation sensor. **(B)** Example of 6 representative individual traces of the intensity of the pHluorin signal within individual vesicles segmented using the mApple fluorescent signal. True degranulation events are confirmed by a sudden surge in pHluorin signal when the pH inside the vesicle is neutralized after fusion with the lumen at the plasma membrane. **(C)** Dual color TIRF time lapse of NK92.mApple-LAMP1-pHluorin expressing cell activated on anti-CD18 and anti-NKp30 antibodies coated glass. The two fluorescent signals are presented separately before the merged panel and the segmented vesicles. Scale bar = 2 µm. **(D)** An entire dataset of NK92.mApple-LAMP1-pHluorin expressing cell activated on anti-CD18 and anti-NKp30 antibodies coated glass has been analyzed to demonstrate the absence of bias between expression level of the degranulation sensor and the probability of observation of degranulation events (N = 11 and 15 cells for DMSO and CK666 treated cells, respectively). Cells were sorted for an intermediate level of expression of the degranulation sensor using the mApple intensity. **(E)** The treatment with CK666 confirmed the same results as obtained in the main figure of the manuscript (Figure 5D). *p* value calculated by unpaired t-test with Welch’s correction two-tailed test. No correlation was observed between the time of the acquisition of the 5 minutes long time lapse for degranulation count and the area of the cell **(F)** or the MFI of the sensor itself **(G)**, confirming that our time window is limited to fully spread NK cells with a mature IS. **(H)** Interestingly, we could reproduce the results of Figure 1G and observe an increase of the number of degranulation events over time in the control (DMSO treated) condition only. **(I)** Finally, the number of degranulation event observed was not correlated with the level of the expression of the sensor.
