# Supplementary material for: Nanoscale Dynamism of Actin Enables Secretory Function in Cytolytic Cells
Source: Curr Biol. 2018 Feb 19;28(4):489–502.e9. doi: 10.1016/j.cub.2017.12.044 (PMC5835143; doi:10.1016/j.cub.2017.12.044)
Supplement: Document S2. Article plus Supplemental Information [file mmc9.pdf]

Current Biology

Article

Nanoscale Dynamism of Actin Enables Secretory Function in Cytolytic Cells

Highlights

- F-actin forms a permissive network at the immune synapse of CTLs and NK cells
- The actin mesh of the mature IS of an NK cell is formed by Arp2/3 branching activity
- Nanoscale actin dynamism is required to allow degranulation
- Nanoscale dynamism is a new opportunity for regulation of cytotoxic function

Authors

Alexandre F. Carisey, Emily M. Mace, Mezida B. Saeed, Daniel M. Davis, Jordan S. Orange

Correspondence

orange@bcm.edu

In Brief

Natural killer cells eliminate target cells via the release of lytic granules into the immune synapse cleft. Degranulation occurs through size-permissive clearances within the cortical actin mesh of NK cells. This Arp2/3-mediated cytoskeleton undergoes constant nanoscale dynamism required to create clearances and achieve cytotoxic function.

Carisey et al., 2018, Current Biology *28*, 489–502
February 19, 2018 Crown Copyright © 2018 Published by Elsevier Ltd.
https://doi.org/10.1016/j.cub.2017.12.044

Current Biology
Article

Nanoscale Dynamism of Actin Enables Secretory Function in Cytolytic Cells

Alexandre F. Carisey,[1,2,3] Emily M. Mace,[1,3] Mezida B. Saeed,[2] Daniel M. Davis,[2,4] and Jordan S. Orange[1,4,5,*]
[1]Center for Human Immunobiology, Baylor College of Medicine and Texas Children's Hospital, Houston, TX 77030, USA
[2]Manchester Collaborative Centre for Inflammation Research, University of Manchester, 46 Grafton Street, Manchester M13 9NT, UK
[3]These authors contributed equally
[4]Senior author
[5]Lead Contact
*Correspondence: orange@bcm.edu
https://doi.org/10.1016/j.cub.2017.12.044

SUMMARY

Natural killer (NK) cells are innate immune effectors that lyse virally infected and tumorigenic cells through the formation of an immunological synapse. Actin remodeling at the lytic immunological synapse is a critical requirement for multiple facets of cytotoxic function. Activating receptor and integrin signaling leads to the regulated turnover and remodeling of actin, which is required for adhesion, sustained receptor signaling, and ultimately exocytosis. NK cells undergo lytic granule exocytosis in hypodense regions of a pervasive actin network. Although these requirements have been well demonstrated, neither the dynamic regulation of synaptic actin nor its specific function, however, has been determined at a nanoscale level. Here, live-cell super-resolution microscopy demonstrates nanoscale filamentous actin dynamism in NK cell lytic granule secretion. Following cell spreading, the overall content of the branched actin network at an immune synapse is stable over time and contains branched actin fibers and discrete actin foci. Similar actin architecture is generated in cytolytic T cells, although the timescale differs from that of NK cells. Individual filament displacement leads to stochastic clearance formation and disappearance, which are independent of lytic granule positioning. Actin dynamism is dependent upon branched network formation mediated by Arp2/3 and contractility generated by myosin IIA. Importantly, the use of small-molecule inhibitors demonstrates that actin dynamism is ultimately needed for granule secretion. Thus, we describe a requirement for nanoscale actin fiber rearrangement in generating the complex actin architecture that enables lytic granule secretion.

INTRODUCTION

Natural killer (NK) cells access cytotoxicity through a series of discrete steps [1] that begin with actin accumulation, continue to polarization of the microtubule organizing center (MTOC) and specialized lysosome-related organelles (lytic granules) toward the immune synapse (IS), and culminate with degranulation onto diseased cells [2–5]. Commitment to target cell lysis follows dominant activation signals in the absence of sufficient inhibitory signaling, after which filamentous actin (F-actin) accumulates at the synaptic interface. Both major cytotoxic lymphocytes, NK and T cells, require actin polymerization and remodeling for lytic synapse formation and function, and human diseases arise from aberrations in this process [6–13]. A pervasive synaptic actin network containing lytic granule permissive clearances has been previously defined in NK cells after their activation, but this work was limited to the observation of fixed samples using several super-resolution microscopy techniques [14–16].

Initial spreading of an NK cell on an activating surface leads to an actin-rich lamellipodium, which is responsive to local signaling [17, 18]. Shortly after, a dense cortical actin mesh is established throughout the IS to fill in the relative void left behind as the lamellipodium progresses outward. Activation-induced NK cell spreading is followed by an actin-dependent movement of signaling microclusters that parallel T cell receptor (TCR) microclusters in T cells [6, 7, 17, 19–28]. Although regions of actin at the NK and T cell ISs are structured differently [14–16, 29–31], indirect evidence in both cell types implies that the malleability of synaptic actin contributes to secretory function. It is unclear, however, whether this is because of large-scale eradication of filamentous actin from the synaptic interface occurring during the lamellipodium stage, or a more complex mechanism taking place within the late and denser actin mesh.

Although initially counterintuitive, as cortical actin may function as a barrier to exocytosis, in many systems the expulsion of organelles or secretory vesicles requires actin-mediated force generation. Therefore, the seemingly contradictory role for actin in secretion, both as a barrier to and as a structural support for degranulation, can be explained by a model in which exocytosis requires the local clearance of cortical actin accompanied by actin-mediated force generation, as recently described in *Drosophila* salivary gland cells [32]. Additionally, the importance of regulating cortical actin density was demonstrated by the impaired NK cell cytotoxicity and viral susceptibility [1, 33] of patients with mutations in the actin remodeling protein Coronin 1A [34]. Despite this, it is impossible to determine whether the presence of large clearances in the actin cytoskeleton alone

This is an open access article under the CC BY license (http://creativecommons.org/licenses/by/4.0/).

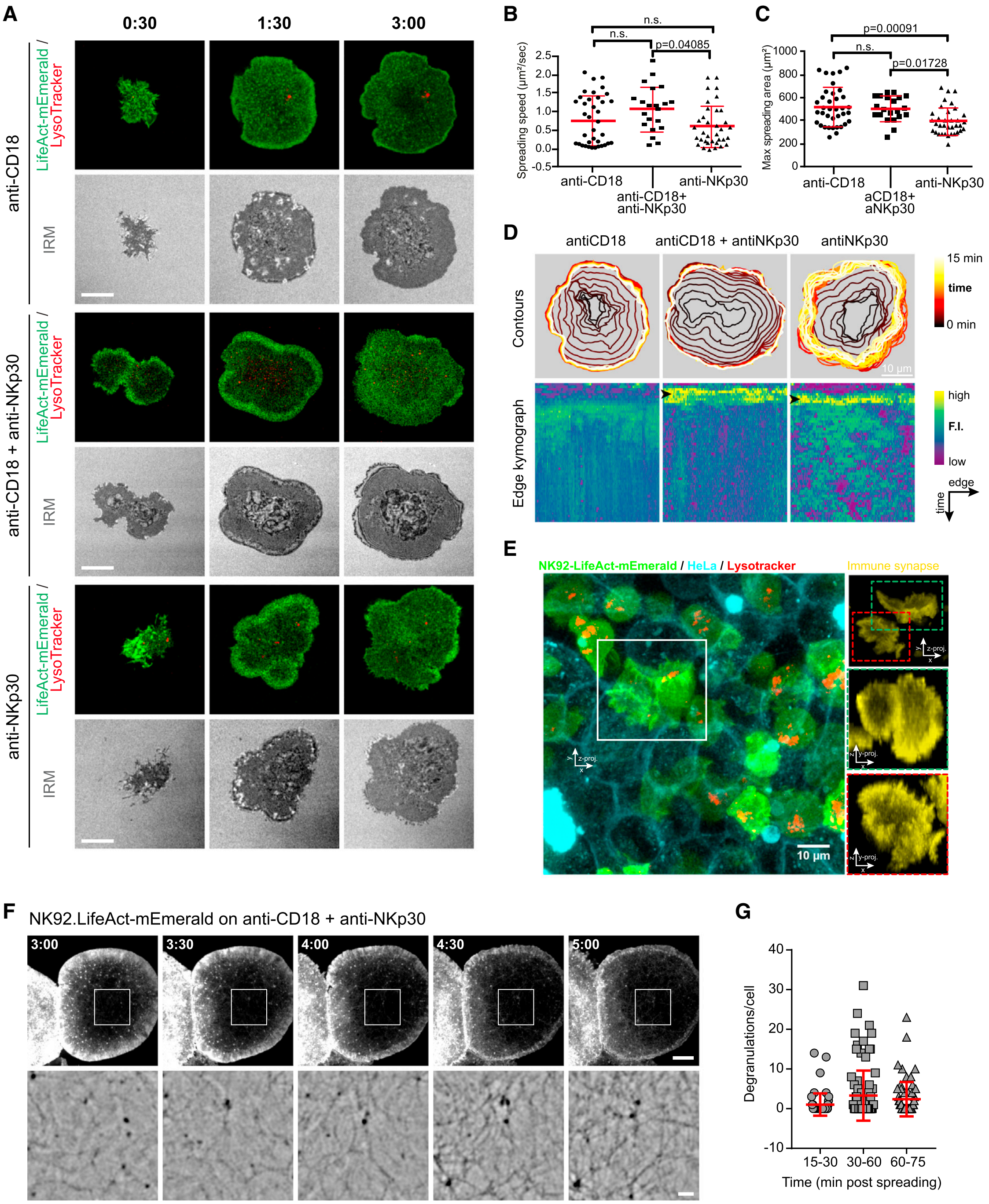

Figure 1. Mature NK Cell Lytic Synapse Is Defined by a Pervasive F-Actin Network

(A) Representative frames from NK92.LifeAct-mEmerald stained with LysoTracker red and seeded on the indicated antibody-coated glass surface and imaged by live confocal microscopy. Scale bars, 5 μm.

(B and C) Spreading speed measured from initial cell contact (B) until the cell footprint reaches its plateau value reported (C). N = 37, 21, and 35 cells, respectively, per condition from 3, 4, and 4 independent experiments. The p value was calculated by one-way ANOVA Kruskal-Wallis test (Dunn's) in (B) and ordinary one-way ANOVA with Tukey's post hoc comparison in (C). n.s., not significant.

(D) Overlay of cell outlines throughout 30 min for each substrate condition (top row); the representative cells are from (A). Each outline was used to define a 2-μm-thick inner rim where the fluorescence intensity (F.I.) of the LifeAct-mEmerald probe was reported in the kymographs below. The small black arrowheads highlight the presence of the actin-rich lamellipodium. Scale bar, 10 μm.

(E) Z projection of a 3D volume from live confocal microscopy of HeLa target cells and NK92.LifeAct-mEmerald effectors (see Movie S1). The IS at the interface between target and effector cells is highlighted in yellow. Two representative ISs (red and green insets) are shown *en face* after a 90° rotation along the x axis (i.e., y axis projection). Representative image of 3 independent repeats. Scale bar, 10 μm.

(legend continued on next page)

represents the key to successful degranulation, or whether the turnover and/or mobility of actin filaments (collectively referred to as "dynamism") are also playing a role in NK cell function. Until now, the existence of, and role for, filamentous actin dynamism in local degranulation has remained hypothetical. Such studies require the direct labeling of actin at the IS, coupled with imaging of sufficient temporal and spatial resolution to address the role of actin in the IS of living cells.

Here we have used multiple super-resolution microscopy techniques to quantify the complex dynamic actin architecture at the NK cell lytic IS in living cells. We find important conserved components of NK and cytotoxic T lymphocyte (CTL) synapses, namely a dynamic synaptic actin meshwork, as well as unappreciated key differences in their kinetics. Whereas the overall architecture of the mature NK cell synapse is stable on a micrometer scale, local dynamism on a nanometer scale is extensive and continuous and occurs through Arp2/3- and myosin IIA-dependent rearrangements of actin filaments. This dynamism is independent of lytic granules at the synapse but is required for cytolytic function through facilitating degranulation. This identifies nanoscale F-actin dynamism throughout the synaptic cortex as a novel, critical regulator of cytolytic function.

RESULTS

Actin Organization at the NK Cell Synapse Begins with a Dual Architecture

To interrogate the organization of actin during the formation of the lytic synapse in living NK cells, we imaged NK92 cells expressing the F-actin reporter (LifeAct-mEmerald) activated by immobilized anti-CD18 and/or anti-NKp30 antibodies [16]. Engagement of either the integrin LFA-1 (CD11a/CD18), the activating receptor NKp30, or both, enables cell adhesion, actin remodeling, and dynamic cell spreading leading to sequential and discrete actin-rich structures, namely a peripheral lamellipodium and a central actin meshwork (Figure 1A). This process is not solely integrin mediated, as the engagement of only the activating receptor resulted in marginally reduced cell-spreading speed (Figure 1B; Figure S1A) and maximum cell area (Figure 1C). However, ligation of the activating receptor (NKp30), with or without integrin co-ligation, caused increased localization of polymerized actin in the periphery of the cell during spreading (Figure 1D, bottom) that was not observed in cells spreading on anti-CD18 alone [18] (Figure S1B). Absence of ligation of the adhesion receptor LFA-1, however, resulted in multiple short-lived lamellipodia (Figure 1A; Figure 1D, top). Additionally, the recruitment of the pool of lytic granules observed here is consistent with previous studies [2, 5], with a greater number of granules present at the synapse after engagement of LFA-1 and an activation receptor. Following the cell spread after any activation (average 240 s), the initial lamellipodium disappeared and the surface occupied by the cell footprint stabilized (Figure 1D, top). The transient initial formation of the thick actin lamellipodial structure in the periphery of the NK cell synapse was also identified in live conjugates between NK92 and HeLa cells and is thus unlikely an artifact of our experimental system (Figure 1E; Movie S1). Although an enriched actin ring was obvious, the central zone contained lower but clearly identifiable densities of F-actin consistent with the actin mesh found in previous studies of fixed cells [14–16].

Using the higher sensitivity and resolution of total internal reflection fluorescence-structured illumination microscopy (TIRF-SIM), we uncovered unequivocally the presence of actin-rich foci and transverse actin fibers within the IS at early time points (Figure 1F; Movie S2) [15]. Actin polymerization occurred rapidly and established the actin mesh, characterized by a visually homogeneous and dense F-actin content at the synaptic cortex. Importantly, NK cells often had uneven plasma membrane topography at the IS, leading to the artifactual observation of F-actin-depleted areas using microscopy techniques with a high Z resolution (Figure 1A, interference reflection microscopy [IRM] channel; Figures S1C and S1D). The center of the IS was never devoid of F-actin, even at the earliest observed time points, and quickly accumulated a dense and persistent actin meshwork within 5 min of activation. Finally, using the degranulation marker LAMP1-pHluorin [16] in NK92 cells, granule exocytosis was observed as early as 15 min; however, degranulation events occurred with the greatest frequency after 30 min (Figure 1G). This is consistent with previous reports of degranulation at the NK cell lytic synapse generally being visualized after 20 min [5].

Thus, integrin ligation and activating receptor signaling provide distinct contributions to generate a complex dual architecture, with a spreading lamellipodium and actin mesh formed by intense F-actin remodeling during IS formation. Additionally, the timing of degranulation indicates that exocytosis occurs through the mature and dense cortical actin cortex rather than the transient actin-depleted interface observed during initial cell spreading.

Granule-Permissive-Sized Clearances Are Sustained in a Persistent F-Actin Mesh at the NK Cell IS

The persistence of the diffuse dense F-actin network was confirmed by stimulated emission depletion microscopy (STED) microscopy in activated NK cells across multiple time points (Figure 2A). To determine the relationship between clearance formation and granule secretion, the frequency of minimally permissive lytic granule-sized actin clearances in fixed NK92 cells activated on anti-CD18 and -NKp30 was measured and found to peak at 40 min (Figure 2B). Similarly, the largest actin clearances suitable for exocytosis of an average-sized 300-nm-diameter granule and above [15] were found to peak at 30 min (Figure 2C). The relationship between actin clearances,

(F) Single frames from live TIRF-SIM microscopy showing the continuous presence of actin fibers throughout the formation and maturation of the IS (from 2 to 5 min following initial contact with the glass). The color scheme of the images within the white square has been inverted, filtered and magnified below to allow the visualization of the dimmest structure while retaining the linearity of the fluorescence signal (see Movie S2). Representative images of 43 cells from 6 experiments are shown. Scale bar, 5 μm (top) and 1 μm (bottom).

(G) Frequency of NK92.LAMP1-pHluorin cells undergoing degranulation detected by TIRF microscopy at the times indicated. N = 60, 67, and 64 cells, respectively, per condition from 3 independent experiments.

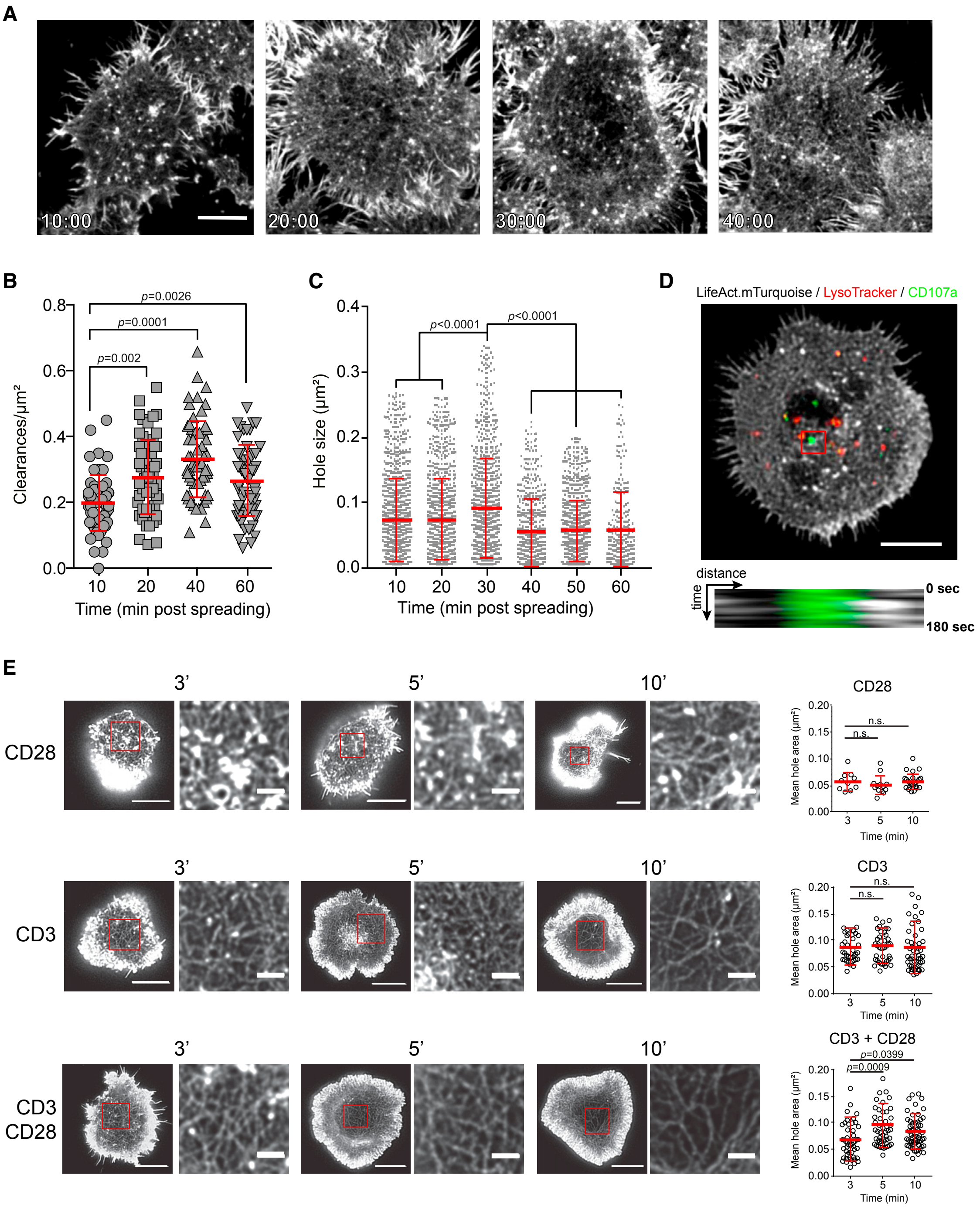

Figure 2. Granule-Permissive-Sized Clearances Persist following Degranulation and Are a Feature of NK and Cytotoxic T Cells

(A) Representative images of NK92 activated on anti-CD18- and anti-NKp30-coated glass for the times indicated, stained for F-actin, and imaged by time-gated STED microscopy. Scale bar, 5 μm.

(B) Quantification of the clearances/μm^2 measured in activated NK92. Each data point represents one cell from N = 58, 59, 59, and 59 cells from 4 pooled independent repeats. The p value was calculated by ordinary one-way ANOVA with Tukey's post hoc comparison.

(C) Quantification of mean hole area measured in fixed activated NK92 cells acquired by SIM at the indicated time points. N = 20 cells per condition; the experiment is representative of 3 independent repeats.

(D) Representative images of NK92.LifeAct-mTurquoise cells showing F-actin (gray), lytic granules (red), and CD107a (green). Scale bar, 5 μm. The kymograph (below) produced from the region highlighted by the red square shown for and showing F-actin (gray) and CD107a (green). Images are representative of 20 cells from 2 independent experiments.

(legend continued on next page)

lytic granule docking, and exocytosis was strengthened by the direct observation of degranulation within a persistent actin clearance, visualized using anti-CD107a (LAMP1) antibody (Figure 2D) [35, 36]. These observations, combined with our data showing degranulation primarily occurring after 30 min of activation at the NK cell IS (Figure 1G), are consistent and in agreement with previous reports indicating that hypodense regions within the actin mesh are sites of lytic granule exocytosis [14–16]. They additionally indicate that NK cell degranulation occurs primarily following spreading and establishment of granule-permissive clearances within a dense actin mesh.

Formation and Maturation of the F-Actin Mesh in T Cells Occur Rapidly

Despite many common steps in their cytotoxic function, CTL and NK cells have marked differences in terms of actin regulation. The highly pronounced thick peripheral ring of actin fibers has been well described in T cells surrounding a central region highly depleted of actin [37–39]. Using STED nanoscopy of primary human T cells fixed at multiple time points after activation, actin meshwork was identified throughout the central zone of the IS on each of three different activating substrates: anti-CD3, anti-CD28, and anti-CD3/-CD28 (Figure 2E). The strongest activation substrates (anti-CD3 and anti-CD3/-CD28) increased mean clearance area measured within the actin mesh similar to NK cells (Figure 2C), albeit more rapidly. In addition, activation on CD3 or CD28 alone, isotype control antibody, or a non-activating poly-L-lysine-coated surface failed to increase clearance area over time (Figure 2E; Figure S2A). To specifically consider the dynamics of F-actin remodeling in major functional CTL subsets, we measured mean individual clearance area (Figures S2E and S2H) and penetrable area (Figures S2F and S2I) in primary human naive ($CD8^+$ $CD45RA^+$; Figure S2D) and memory ($CD8^+$ $CD45RO^+$; Figure S2G) CTLs. Both demonstrated increased granule-permissive-sized clearances within the meshwork after activation, which again appeared sooner, peaked earlier, and were shorter lived compared to those in NK cells. The clearances, however, were still detected, albeit at a lower frequency, at 30 and 40 min following activation. In contrast, in primary NK cells, granule-permissive-sized clearances were robustly maintained up to 40 min after activation through LFA-1 and NKp30 in both fresh (Figures S2J–S2L) and interleukin-2 (IL-2)-activated cells (Figures S2M–S2O). Interestingly, the addition of IL-2 led to a global 4-fold increase of the granule penetrable area (Figures S2L and S2O) without having an impact by itself (in the absence of NKp30 engagement; first plot in Figure S2O). To address whether the kinetics of IS formation were faster in T cells, actin was labeled with silicon rhodamine conjugated jasplakinolide derived dye (SiR-actin) and lytic granules by LysoTracker red in primary $CD8^+$ $CD45RO^+$ T cells. Cell spreading on anti-CD3/-CD28 was markedly faster (Figure S2B), with a maximum cell footprint area established within 2 min (as opposed to 4.5 min for NK92 cells; Figure S1A). Notably, even at this early time point in T cells, the maximum amount of lytic granule signal was already detected at the plane of the IS (Figure S2C). Together, these data identify the actin meshwork as a conserved characteristic of T and NK cell lytic synapses, while underscoring important differences in their kinetics.

Actin at the NK Cell Synapse Is Stable yet Locally Dynamic

To define the dynamics of actin in NK cell lytic synapses, we performed TIRF-SIM on live activated NK92 expressing LifeAct-mEmerald. We detected no significant retrograde movement of F-actin in the lamellipodia (Figures 3A and 3B; Movie S3), and the amount of F-actin was stable (Figure S3C) during synaptic maturation, which began after the cell footprint had stabilized (Figure 3A; Figure S3B). The possibility of reconstruction or illumination artifact from the use of TIRF-SIM was excluded by using STED microscopy, which identified similar properties of both actin area and content (Figures S3C and S3D). Detailed kymograph analysis followed by the extraction of the forward, backward, and stable components (Figure 3C) confirmed that, in this model of NK cell activation, no persistent retrograde flow is observed, unlike in T cells. Using optical flow analysis on several regions of the cell edge after the formation of the IS, we could only detect short sequences of outward actin polymerization that were concomitant with membrane protrusion (Figure S3E; Movie S3), another major difference with the retrograde flow described in T cells.

Although globally stable, at the nanoscale level of the actin network, the activated NK cell lytic synapse was pervasive but contained granule-permissive-sized clearances (Figure 3D). The critical use of the TIRF-SIM technique allowed us to achieve an unmatched level of resolution at high signal-to-noise ratio and explore any nanoscale actin dynamics and clearance segmentation. The actin fiber mesh was detected [15] and used as a mask to identify the clearances; fibers were then reconstructed using orientation filter transform (OFT) filament analysis [40] (Figure S3J; Movie S4). Actin fibers did not exhibit unified directional or radial flow (Figure 3D, optical flow panels; Movie S4) but instead demonstrated nanoscale dynamism, creating local opportunities for appearances and disappearances of clearances (mapped in the clearances panels in Figures 3D and S3J). Plotting the size of clearances above 0.5 μm^2 reveals a large distribution of area (up to 3 μm^2) nested within the dense cortical actin structure present at all time points (Figure 3E) but rapidly evolving within the frame rate of this live-cell experiment (Figure 3F). This constant cycle of opening and closing of clearances was confirmed by STED microscopy (Figure S3F). When imaged at 1- and 10-s time intervals by STED microscopy, variation in the frequency of granule-permissive-sized clearances was observed (Figures S3G–S3I). At the faster frame rate, we observed clearances that were maintained over multiple frames of imaging. There was, however, still permanent variation in the frequency of clearances, illustrating that even within short

(E) Primary human T cells were isolated from peripheral blood of healthy donors and activated on anti-CD3, anti-CD28, or anti-CD3/-CD28 for the times indicated prior to fixation and staining with phalloidin Alexa Fluor 488. Cells were imaged by time-gated STED microscopy. The regions inside the red boxes are magnified on the right of each panel. Scale bars, 5 μm and 1 μm (insets). Mean hole area was measured and is shown in the graph (right) of each condition. N = 10, 13, 24, 37, 41, 50, 49, 50, and 61 cells, respectively, pooled from 3 independent repeats. The p value was calculated by one-way ANOVA Kruskal-Wallis test (Dunn's). Similar analysis was performed in primary T $CD8^+$ and NK cells in Figure S2.

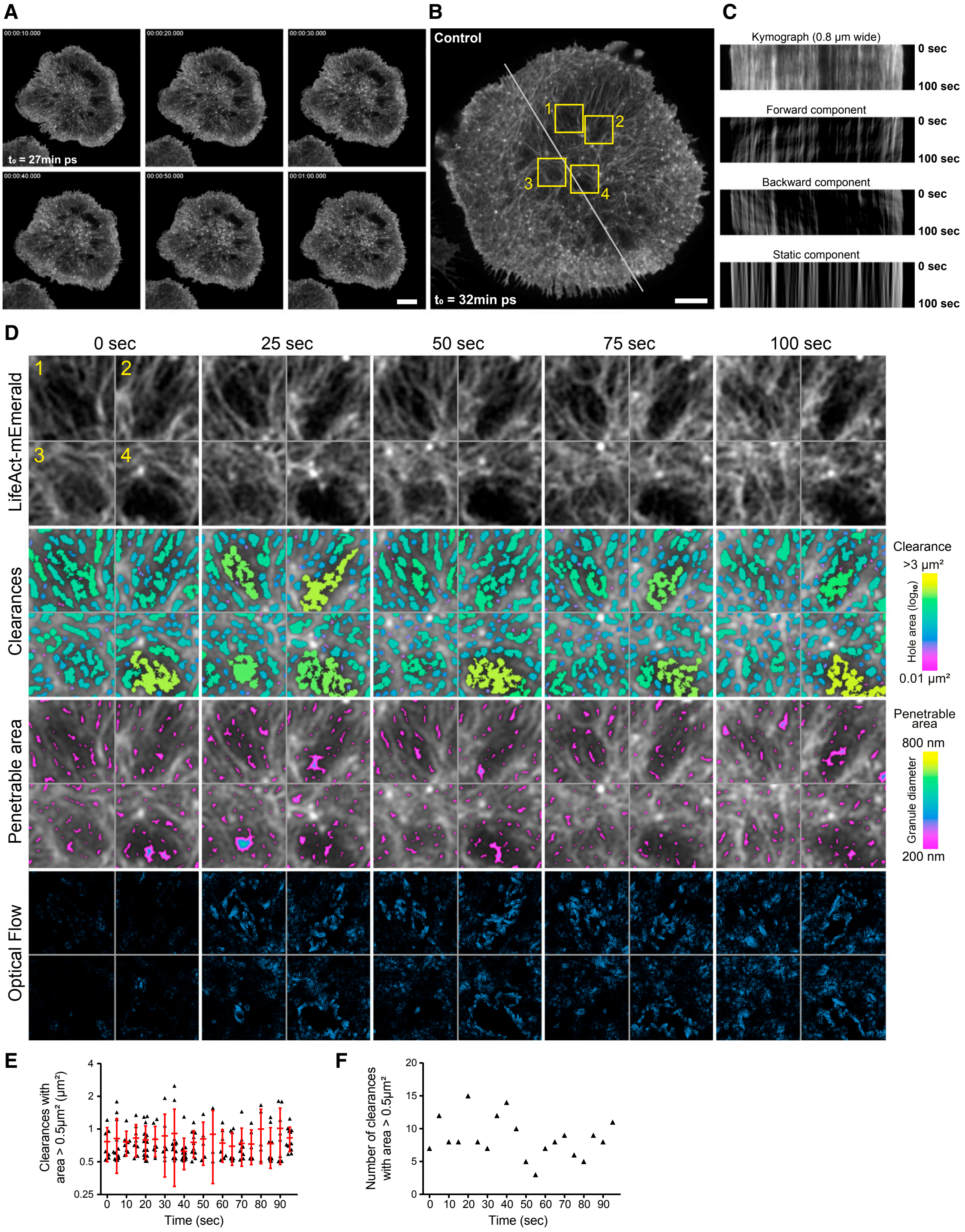

Figure 3. Global Actin Stability at the Mature NK IS Is Coupled to Local F-Actin Dynamism

NK92.LifeAct-mEmerald cells were activated for 20 min prior to initiation of imaging by TIRF-SIM microscopy at 5-s intervals for 5 min. Scale bars, 5 μm. ps, post-spreading.

(A) Time-lapse series of a single cell representative of 43 cells from 6 independent experiments imaged showing stable actin level at the IS (see Figures S3A–S3D).

(B) Single frame of a time-lapse series of a representative cell (see Movie S5, left) with a transversal line indicating the axis used for the kymograph analysis in (C).

(C) Kymograph analysis of the LifeAct-mEmerald signal measured for 100 s (optical flow analysis; see Movie S3). The total signal is plotted on the top and the forward; backward and static components are below highlighting the stability of the edge regions of the mature NK IS (imaged 32 min post-spreading on the activating surface).

(D) Visualization of the nanoscopic dynamism within the cortical actin cytoskeleton in 4 regions indicated in their context in (B). The first row details the LifeAct-mEmerald signal over time. Below, the area of segmented clearances is color coded according to the color scale (right) and further analyzed below by indicating

(legend continued on next page)

timescales, actin filament movement creates clearance dynamism. When cells were treated with jasplakinolide, a commonly used actin-filament-stabilizing drug, the lateral movements and reorganization of the actin filaments abruptly stopped despite the overall contraction of the actin mesh due to enhanced activity of myosin II (Figure S3K; Movie S5). As a control experiment, we verified that the treatment of NK92 with jasplakinolide (and all other inhibitors used in this study) was not significantly affecting the viability of the cells (Figure S4A).

Upon close inspection, small actin-rich foci displaying limited mobility could be distinguished within the synaptic mesh and corresponded to nodes at the crossroad of multiple actin fibers (Figure 4A; Movie S6). These foci demonstrated no preferential location or concerted centripetal flow (Figure 4A), and could appear, disappear, fuse, or split (Figure 4B; Movie S6). Measurement of foci lifetime demonstrated that, although there was significant variability in the time that foci were present, their average lifetime is 42 s (±43 s), although a small proportion (3.06%) are more stable and persisted for many minutes, exceeding the total duration of our super-resolved imaging (Figure 4C). To evaluate their dependence on the Arp2/3 branching complex, foci were enumerated in NK92 cells treated with the Arp2/3 inhibitor CK666 [41, 42], fixed, and stained with phalloidin after establishment of the IS. Arp2/3 inhibition completely abrogated the actin foci (Figure 4D), which supports their role in forming and maintaining the multinodal network architecture via Arp2/3-mediated filament branching. Treatment with the small inhibitor CK666 followed by a washout of the drug performed on the actin reporter cell line NK92.LifeAct-mEmerald demonstrated the rapid disassembly of the actin foci within 45 s post-treatment (Figure 4E; Movie S7) and their steady recovery occurring as early as 60 s after removal of CK666 from the imaging medium. Interestingly, we could marginally observe on this live-cell dataset a thickening of the actin fibers (Figure S4B) despite an overall loss of actin recruited at the synapse (Figure S4C). To complement the results obtained with the Arp2/3 inhibitor, we treated NK92 cells spread for 20 min with 50 μM pan-formin inhibitor SMIFH2 [43] for 10 min before imaging them during the washout of the drug. Because SMIFH2 is phototoxic, we could only compare the actin level prior to and after the treatment with one single time point acquired during the incubation with the compound. We could not identify a significant consequence on the recruitment of actin at the synapse (Figure S4D) or its general architecture (data not shown) after pan-formin inhibition.

Overall, extensive F-actin dynamism is present and responsible for rapid turnover of local actin clearance. This defines local F-actin dynamism as the source of an active, motile actin meshwork that is permissive for the access of lytic granules to the plasma membrane.

Lytic Granule Exocytosis Requires F-Actin Dynamism

Although Arp2/3 is localized to the NK cell IS, required for IS maturation [44], and supports the formation of the actin foci described above, its role in the maintenance of the mature IS has not been directly tested. We treated NK cells with CK666 following activation and after the mature IS had formed and found that the actin mesh adopts a different morphology with unbranched actin filaments (Figure 5A). Despite a significant increase in the frequency of large clearances (Figure 5B; Figure S4B) and the docking of granules onto the actin mesh (Figure 5C), degranulation was significantly impaired (Figure 5D; Figure S5). Live-cell STED microscopy of similarly treated cells confirmed that Arp2/3 inhibition depleted branched actin, leaving long, bundled actin filaments at the NK cell synapse (Figure 5E) that lacked dynamism (Figure 5F). Furthermore, stabilization of the F-actin network using jasplakinolide in activated NK cells also decreased degranulation events compared to controls (Figure 5D). Thus, there is a critical role of Arp2/3-mediated synaptic actin dynamism, not just in lytic synapse formation and clearance structuring (Figure 4) but also in lytic granule secretion (Figure 5).

In NK cells, myosin IIA is localized to the activating synapse, and the loss of myosin IIA function through small-molecule inhibition, or naturally occurring mutations in *MYH9*, leads to significantly reduced NK cell cytotoxicity [45–47]. Whereas formation of the IS is seemingly unperturbed in these instances, exocytosis is impaired. When evaluated on a nanoscale, however, treatment of NK92 with blebbistatin after activation and IS maturation led to an increased density of fibers resulting in fewer granule-permissive-sized clearances despite retention of the overall actin mesh structure (Figures 5G and 5H) without any significant increase of the total amount of actin at the synapse (Figure S4E). Functionally, blebbistatin treatment led to reduced degranulation (Figure 5D) despite the delivery of lytic granules to the synapse as detected by TIRF microscopy (Figure 5I). This emphasized a novel role for myosin IIA in the maintenance of F-actin clearances with an area permissible for the passage of lytic granules to access the plasma membrane.

Thus, actin dynamism generates an activated steady state of cortical actin fibers that includes active remodeling and contraction, both of which are required for secretion needed for cytolytic function.

Actin Dynamism Is Independent of Lytic Granule Appearance and Disappearance

As lytic granules are motile prior to settling into regions of F-actin hypodensity [9] yet actin clearances are dynamic, we sought to determine whether the presence of granules was directly influencing local actin dynamics. To determine the effect of lytic granule arrival or exit on synaptic actin, we activated NK92.LifeAct-mTurquoise cells with LysoTracker red-labeled granules for 30 min and imaged by STED microscopy with greater axial resolution than TIRF. We analyzed the movement of F-actin at the sites at which LysoTracker-labeled granules appeared or disappeared by kymograph or line profile analysis. Actin clearances were observed prior to the arrival of granules (Figure 6A, top) or maintained following degranulation (Figure 6A, bottom). This was also illustrated by kymograph analysis of cells in which lytic granules appeared or were stationary (Figure 6B).

the clearances compatible with the extrusion of granules with size ranging from 200 to 800 nm (see Figure S3J and Movie S4). The last row visualizes the dynamism surrounding the sites of clearance formation as a result of filament rearrangement using optical flow mapping.

(E and F) Plot (E) and count (F) of the area of the clearances above 0.5 μm^2 over the time course of the super-resolved time lapse (see Movie S5). Clearances continuously appear and disappear on a short timescale within the cortical actin mesh at the mature IS of NK cells.

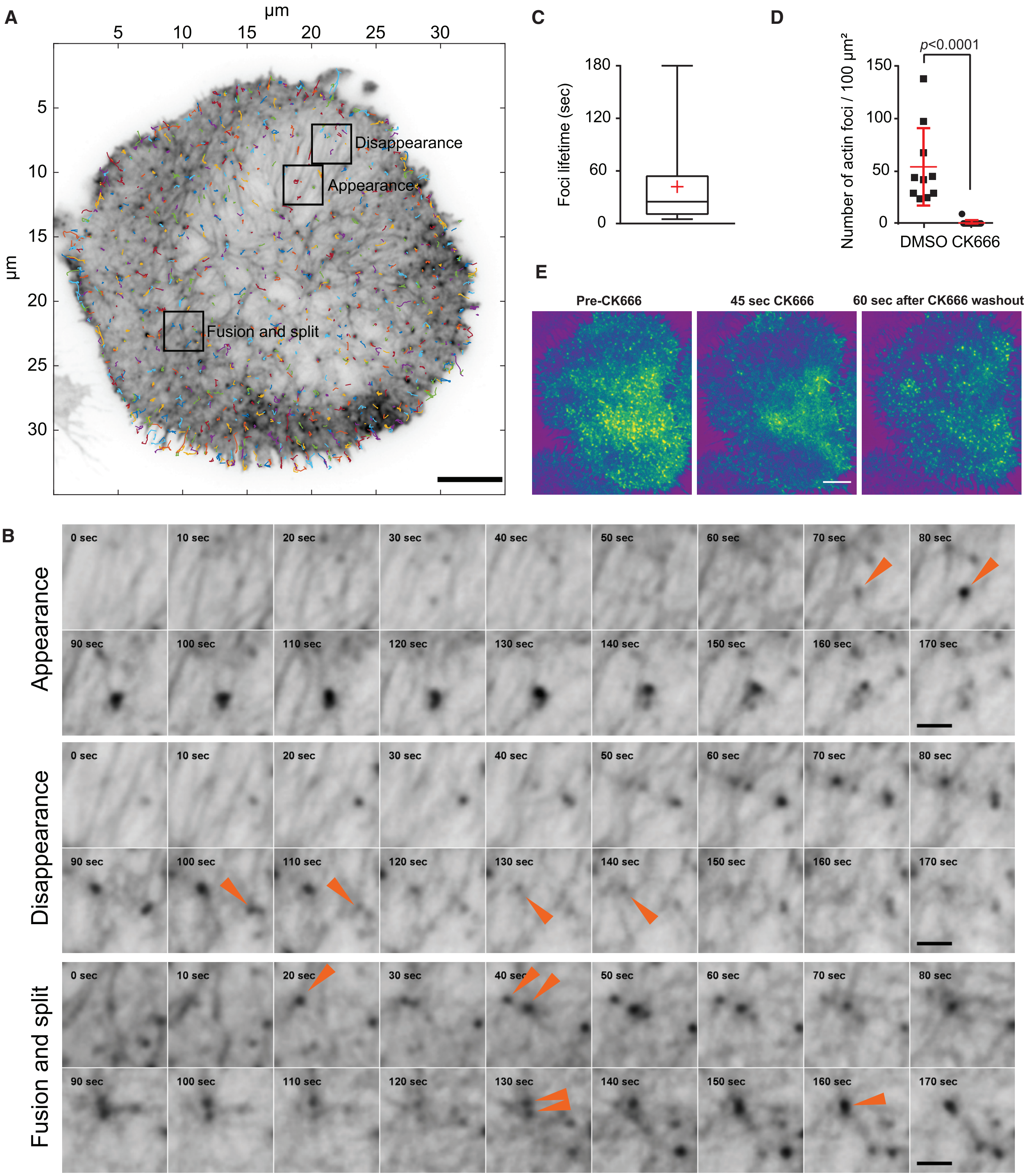

Figure 4. Foci Are Present at Sites of Local Actin Rearrangement

(A and B) Representative NK92.LifeAct-mEmerald cell activated (A) (inverted color from Figure 3B) for 20 min on anti-CD18 and anti-NKp30 prior to imaging by TIRF-SIM including F-actin foci that undergo dynamic behaviors highlighted by the orange arrowheads in the time series in (B) (see Movie S6). Colored tracks indicate their displacement over 95 s. Scale bars, 5 μm (A) and 1 μm (B).

(C) Quantification of the lifetime of the actin foci. Data are pooled from 20 cell averages at 42 s ± 43.3 s. Additionally, a small proportion (3.06%) of all actin foci measured have a turnover longer than the acquisition of the time lapse (>3 min).

(legend continued on next page)

Locally, filament dynamics were maintained over time, and even after granule disappearance (Figure 6B, bottom), demonstrating that each event is independent of the other. Thus, we conclude that the random creation and maintenance of clearances caused by constant F-actin dynamism on a nanoscale are independently regulated from lytic granule delivery and presence, yet required for their exocytosis needed for cytotoxic function.

DISCUSSION

Polymerization, remodeling, and regulation of F-actin are critical for lytic IS formation and function [48], and defects result in human disease [49]. Although it has previously been shown that NK cell degranulation occurs within regions of actin hypodensity at the lytic synapse [14–16, 50], the dynamic properties of this network were unknown, as was any role for actin dynamics in exocytosis required for lytic function. A significant challenge in the field has been combining sufficient temporal and spatial resolution to gain insight into the molecular events that occur at the lytic IS. The use of lipid bilayer or functionalized glass has enabled high- and super-resolution imaging to provide key insight into the cytoskeletal and molecular events that accompany lytic IS formation and function. However, high-intensity illumination and limited temporal resolution of these approaches make them difficult to apply to highly dynamic cytoskeletal events. The recent development of the lattice light sheet has enabled unprecedented temporal resolution combined with improved spatial resolution over conventional confocal imaging; however, nanoscale structures visualized by fixed-cell super-resolution may not be detectable using this method, particularly F-actin [51]. Here, we overcome technical limitations from previous studies using fixed cells [14–16] to report the architecture of the actin cytoskeleton on a nanoscopic scale using live-cell super-resolution microscopy approaches. We have used multiple imaging modalities (STED, SIM, TIRF, and TIRF-SIM) to take full advantage of the strengths of each system and reproduce our data across multiple platforms. We also recapitulate the NK cell lytic IS with living target cells to corroborate our data shown using higher-resolution imaging done on functionalized glass. Although modeling the lytic IS between two living cells with sufficient resolution remains a formidable and open challenge in the field, we have attempted to combine thoughtful modeling with multiple imaging modalities to understand the nature of nanoscale actin dynamism at the lytic IS.

De novo actin polymerization is an early step in the formation of the IS leading to cytotoxicity. Although both NK and T cells form a lytic synapse dependent upon actin, there has not been a direct comparison of the two with enhanced spatiotemporal resolution. Traditionally, the T cell synapse has been described to have a ring of cortical F-actin surrounding an actin-depleted center [37–39] with symmetrical retrograde actin flow [25, 28, 29] after activation leading to a central "sink." Recent work using super-resolution microscopy of the IS of T cells has challenged this view and described a ramified actin network below the typical resolution achievable by standard microscopy [30]. The F-actin flow from the periphery is needed for TCR microcluster formation and movement that is in turn required for activation signaling [7, 22, 26]. The actin architecture at the T cell synapse exists in discrete regions that have been proposed to correspond to a lamellipodium, rich in Arp2/3-dependent branched actin networks, and a lamella [31] that sits behind the lamellipodium. Here we show the formation of an enriched actin ring, similar to a lamellipodium, surrounding the IS in NK cells during their initial contact formation and spread, followed by the delayed enrichment of F-actin in the central zone of the synapse. Importantly, we demonstrate the existence of a cortical actin network at the IS with variable clearance size range in response to specific activation signal in T cells. The actin-rich foci that we observe at the NK cell IS resemble those previously described in T cells, yet their lack of centripetal motility and reduced size differentiate them from the dot-like structures supporting T cell microclusters [27]. They are more closely related to the actin vortices described in Fritzsche et al. [52], because they rely on Arp2/3, are similar in size and localization, and are not sensitive to myosin inhibition. The reduced lifetime observed in the actin foci of NK cells compared to the actin vortices of HeLa cells could be explained by a generally higher turnover of actin within the IS. This hypothesis could be further explored by analyzing local actin turnover using fluorescence recovery after photobleaching (FRAP) methodology or quantitative fluorescent speckle microscopy (qFSM) [53]. The most striking difference between NK and T cells is in the kinetics of synapse formation leading to a later range of times for degranulation in NK cells when compared to T cells [51]. Additionally, although we detected symmetrical lamellipodia formed following NK cell spreading and activation, they essentially disappeared after IS maturation. We show that, although activating receptor ligation permits spreading, integrin engagement stabilizes the footprint, leading to a larger, more symmetrical synapse containing a dense dynamic actin mesh. Furthermore, activating receptor ligation initiates the formation of granule-permissive actin clearances that could be used by lytic granules to transit through a cortical mesh otherwise over-reticulated prior to exocytosis [14–16, 50].

Centrosome polarization to the synapse delivers lytic granules directly, leading to lytic granule docking and degranulation [39, 51]. Our data suggest that these events are coincident with the generation of a dynamic, permissive actin network that enables degranulation. We propose that the nuanced actin architecture at the lytic IS reflects additional regulatory checkpoints as a precautionary measure for the lack of antigen restriction in NK cells [54, 55] to restrain and regulate a lethal force. This hypothesis is supported by the greater mean time to both granule polarization and killing of the target in NK cells than

(D) Quantification of the number of F-actin-rich foci in NK92 cells activated for 10 min prior to administration of 50 μM CK666 for an additional 20 min. Images were acquired by time-gated STED microscopy, and the number of F-actin foci present at the synapse were measured using image segmentation. N = 10 and 13 cells, respectively, from 1 experiment representative of 4 experiments. The p value was calculated by Mann-Whitney unpaired two-tailed test.

(E) Representative live-cell imaging experiment using TIRF-SIM where NK92.LifeAct-mEmerald cells have been plated on an antibody-coated surface and imaged through the treatment and washout of 50 μM CK666. The actin foci are sensitive to inhibition of CK666 but recover shortly after washout of the compound (see Movie S7). The cell is representative of 12 cells from 4 independent repeats. Scale bar, 5 μm.

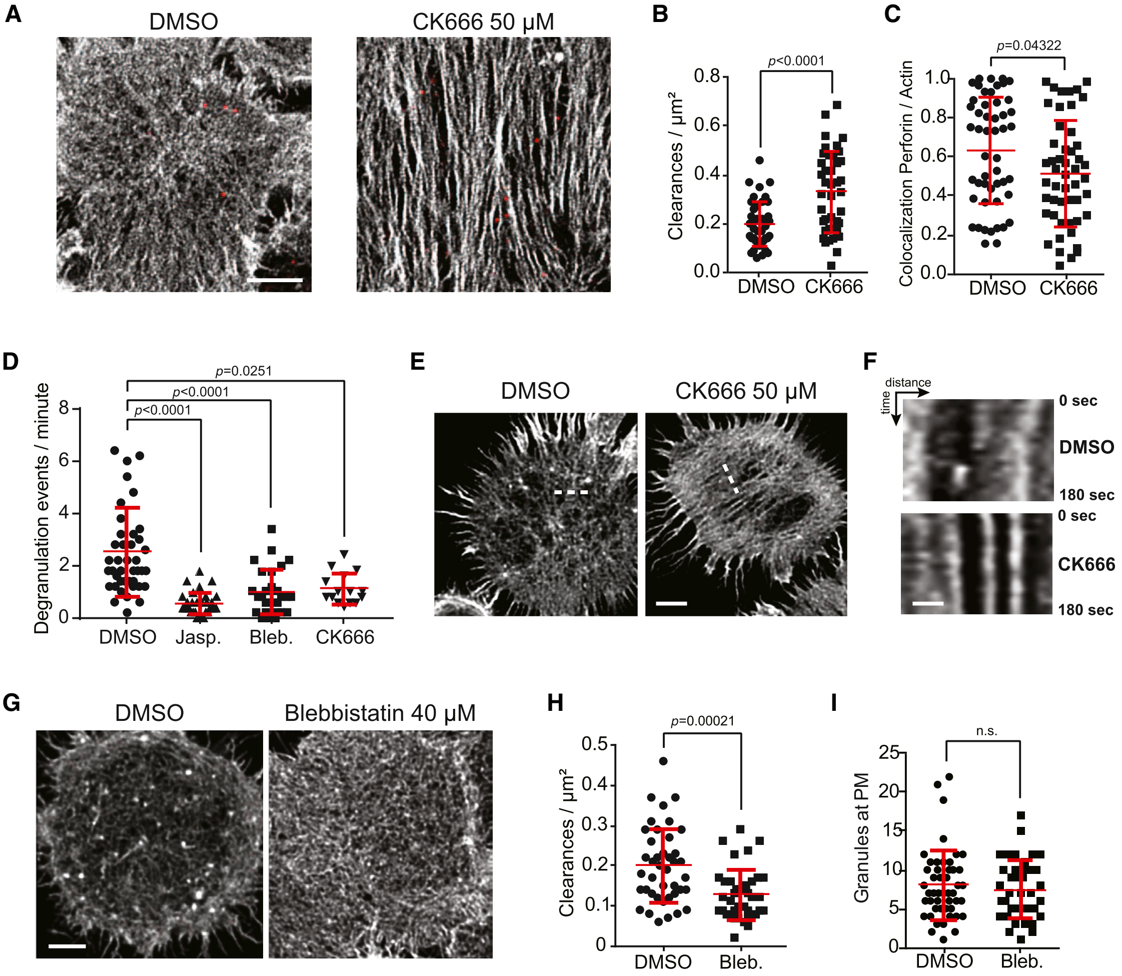

Figure 5. Maintenance and Dynamism of Actin Clearances at the Lytic Synapse Allow Degranulation

(A) F-actin (gray) and perforin (red) staining acquired by STED microscopy of NK92 cells activated on anti-CD18- and anti-NKp30-coated glass for 20 min prior to addition of 50 μM CK666 or DMSO for 10 min. Shown is one representative cell from each condition of greater than 100 cells per condition from 4 independent experiments. Scale bar, 2 μm.

(B) Frequency of granule-permissive clearances was calculated from cells acquired as in (A). N = 41 per condition from 2 experiments pooled representative of 4 experiments performed. The p value was calculated by unpaired t test with Welch's correction two-tailed test; outliers were removed by robust regression and outlier removal (ROUT) (1%).

(C) Co-localization between perforin and phalloidin at the site of each granule for cells acquired as in (A). Each data point represents a single granule. N = 50 per condition from 2 pooled independent experiments representative of 4 repeats. The p value was calculated by Mann-Whitney unpaired two-tailed test; outliers were removed by ROUT (1%).

(D) Live-cell time lapses of NK92 cells expressing mApple-LAMP1-pHluorin were acquired by TIRF following 20 min of activation and 10 min of treatment. Degranulations were measured as explained in STAR Methods (Figure S5). The number of events of degranulation observed per min is reported with one cell for each data point. N = 41, 28, 31, and 15 cells, respectively, pooled from 3 independent repeats. The p value was calculated by one-way ANOVA Kruskal-Wallis test (Dunn's); outliers were removed by ROUT (1%).

(E) NK92.LifeAct-mTurquoise cells activated as above and image series captured by live-cell STED following 10 min of CK666 treatment. Representative images from 24 cells from 4 experiments are shown. Scale bar, 2 μm.

(F) Kymograph analysis using a line profile of the regions indicated in (E). Scale bar, 0.5 μm.

(G) NK92 cells treated with blebbistatin, stained for F-actin, and imaged by fixed-cell STED microscopy. Scale bar, 2 μm.

(H) Cells were quantified for the presence of granule-permissive-sized clearances. N = 41 and 38 cells, respectively, from 2 pooled independent experiments representative of 4 repeats (see Figure S4B). Each data point represents a single cell. The p value was calculated by Mann-Whitney unpaired two-tailed test; outliers were removed by ROUT (1%).

(I) Cells treated with blebbistatin and imaged by live-cell TIRF microscopy were enumerated for the number of granules in the TIRF field as detected by LysoTracker staining. N = 48 and 39 cells, respectively, from 2 pooled independent experiments representative of 4 repeats. The p value was calculated by Mann-Whitney unpaired two-tailed test; outliers were removed by ROUT (1%). PM, plasma membrane.

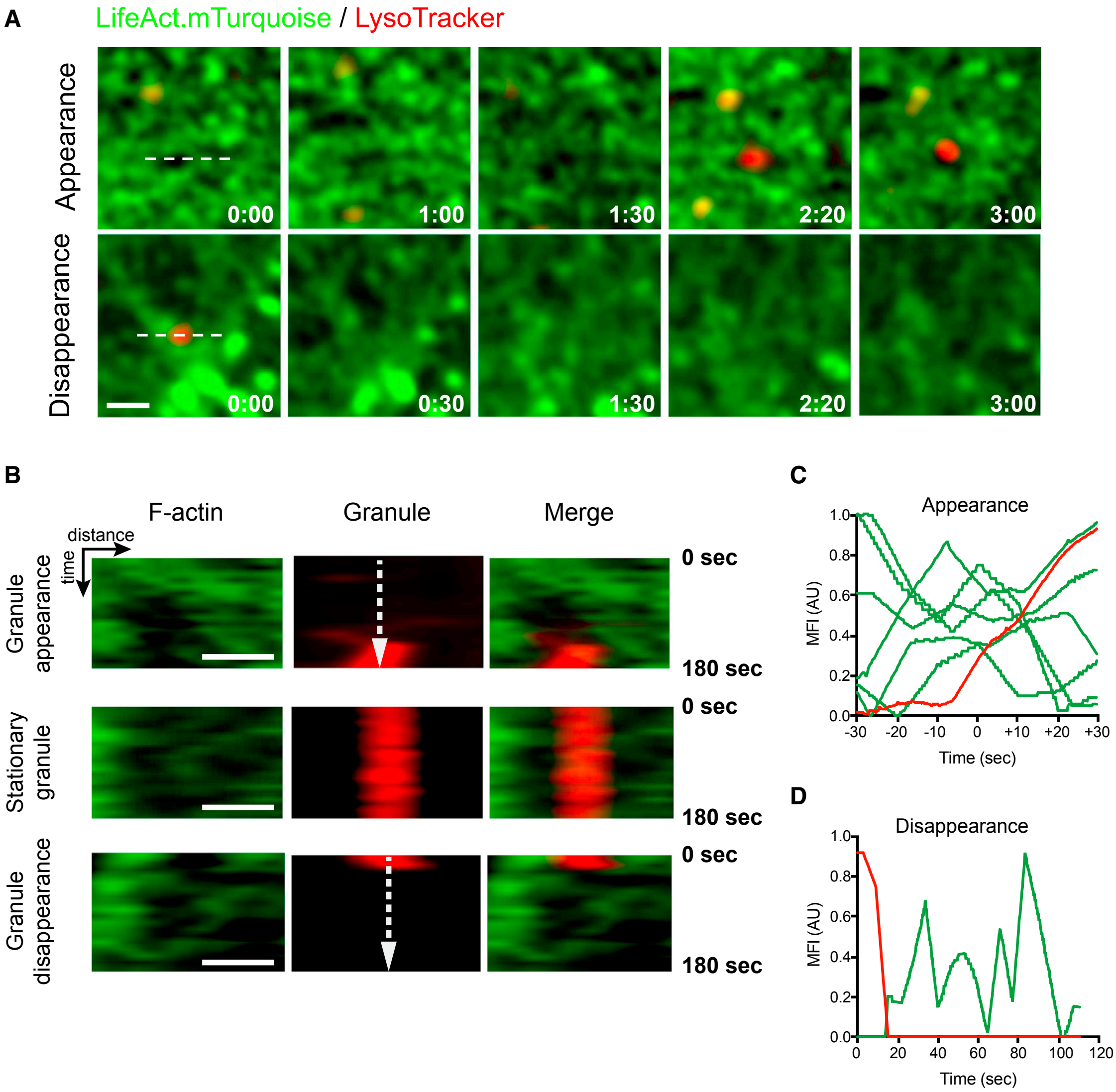

Figure 6. Actin Filament and Lytic Granule Movement Occur Independently

NK92.LifeAct-mTurquoise cells were labeled with LysoTracker red prior to activation on anti-CD18- and anti-NKp30-coated glass for 20 min and then imaging of single cells at 10-s intervals by STED microscopy.

(A) Representative cells highlighting a 3 × 3 μm region of interest that includes the capture of a granule appearance (top) or disappearance (bottom) during the time of imaging. Scale bar, 1 μm.

(B) Kymograph analysis using a line profile of the regions indicated in (A) (x axis) measured over time (y axis). Three situations are illustrated: granule appearance in an existing clearance (top), stationary granule over dynamic actin filaments (middle), and granule disappearance through a closing clearance (bottom). Scale bars, 0.75 μm.

(C) Line profile analysis of granule appearance shown by the vertical dashed line shown in (B). The mean of 6 granules is shown normalized to their arrival in the imaging field of view (one example of a granule intensity profile is in red). Individual line profiles of F-actin over the granule are shown in green. MFI, mean fluorescence intensity.

(D) Individual line profile (dashed vertical line) across the kymograph of the disappearing granule shown in (B). Data are representative of 24 cells from 3 independent experiments.

CTLs, and the previous observations that the strength of TCR signaling can affect the speed of granule delivery and release in T cells [51, 56, 57]. Our results showing the presence of actin fibers throughout T cell and NK cell synapses also support the hypothesis that regulated exocytosis of lytic granules requires actin-generated force. This is in accordance with the observation that sites of degranulation are marked by local force exertion mediated by non-muscle myosin in T cells [58] and ephemeral synaptic granule persistence in NK cells [9]. Our use of inhibitors lends insight into the specific roles for Arp2/3 and myosin IIA in the regulation of actin architecture following IS formation. Whereas continuous inhibition of Arp2/3 complex function prevents NK cell adhesion to targets and synaptic F-actin assembly [44], our delayed inhibition of Arp2/3 only after the synapse has matured demonstrates it is required to maintain the filament and foci-based [52] actin network architecture to allow

exocytosis, without affecting the delivery of lytic granules to actin clearances. Our data do not distinguish whether the requirement for Arp2/3 function is in the continuous displacement of actin filaments to facilitate granule passage through cortical actin or as a scaffold for the contraction force required to promote exocytosis. Nevertheless, the complete abrogation of degranulation after CK666 treatment, despite larger clearance areas, suggests that the role of Arp2/3 is not limited to the formation of granule-permissive-sized clearances but includes the polymerization of actin structures that promote lytic granule extrusive force [9]. The disparate requirement for formin that we measured may reflect differing timescales for the formation, establishment, and function of the T and NK cell lytic synapses. Hence, the absence of phenotype following the treatment of the mature NK cell synapse with the pan-formin inhibitor could testify to distinct primary mechanisms of actin homeostasis/rearrangement at different points in synapse evolution, essentially suggesting a functional uncoupling between synapse formation and synapse maintenance in NK cells. Nevertheless, the high dependency on Arp2/3-mediated actin fibers and the limited role of formin-mediated actin remodeling in the mature synapse of NK cells are consistent with the work recently published on the mature T cell synapse [30]. Collectively, these data emphasize that the maintenance of F-actin dynamism after activation represents a key opportunity for regulating the cytolytic process.

Stabilization of actin filaments using jasplakinolide should not affect the activity of motor proteins, yet we found that it strongly suppressed degranulation. Thus, intrinsic renewal of the F-actin scaffold itself is required for the lytic granules to cross the network of cortical actin. Myosin IIA is required for human NK cell function and in localized lateral force exertion at sites of exocytosis in CTLs [58], although not for conjugate formation or lytic granule polarization to the IS [45–47]. Here we show that myosin IIA inhibition led to a denser synaptic actin mesh with fewer granule-permissive-sized clearances, which does not allow degranulation. Therefore, in addition to its role in lytic granule movement, myosin IIA provides lateral contractility of the synaptic F-actin mesh to maintain dynamic granule-permissive-sized actin clearances required for degranulation.

The current study is the first to demonstrate the constant rearrangement of the F-actin network at the IS in the context of cytolytic function and identify Arp2/3- and myosin IIA-mediated F-actin dynamism as a critical component to secretory function needed for cytotoxicity. Our parallel observation of F-actin, lytic granules, and degranulation events demonstrates complex and non-linear relationships, as arrival of a granule at the IS did not trigger the systematic formation of a larger clearance or precede compulsory degranulation. Although our system did not enable us to unambiguously define exocytic events, we sought to image granules that we were confident had not left the field of imaging due to being retracted or displaced. Together with our independent measurements of degranulation and actin clearance formation, these data suggest that rapid closing of cortical actin does not follow NK cell degranulation, as has been recently described for T cells [59]. Given the significant period in which NK cell lytic granules undergo movement at the IS following their delivery [9], we hypothesize that actin dynamism is uncoupled from granule delivery but serves to increase the probability of a stochastic encounter between a lytic granule and a granule-permissive-sized clearance. We propose nanoscale dynamism as an additional regulatory component for the transfer of lytic granules across the cortical actin mesh at the IS. The emphasis on the nanoscopic scale is supported by our data opposing the overall stability of the actin content over time while intense reorganization occurs at the filament level. The central requirement for dynamism is demonstrated by the suppressive effect of actin-stabilizing drugs on the degranulation rate. Together, these data define nanoscale dynamism of F-actin at the IS as a prerequisite for secretion and lytic function.

STAR★METHODS

Detailed methods are provided in the online version of this paper and include the following:

- KEY RESOURCES TABLE
- CONTACT FOR REAGENT AND RESOURCE SHARING
- EXPERIMENTAL MODEL AND SUBJECT DETAILS
  - Cell lines and cell culture
  - Primary cell isolation
- METHOD DETAILS
  - Plasmids and transfection
  - Sample preparation for microscopy
  - Confocal and STED microscopy
  - TIRF microscopy live imaging acquisition
  - Cell viability assay following drug treatment
  - Fixed SIM and live TIRF-SIM microscopy
  - Parameters of live cell imaging experiments
  - Image analysis
  - Figure preparation
- QUANTIFICATION AND STATISTICAL ANALYSIS

SUPPLEMENTAL INFORMATION

Supplemental Information includes five figures and seven movies and can be found with this article online at https://doi.org/10.1016/j.cub.2017.12.044.

ACKNOWLEDGMENTS

The authors wish to thank Drs. Kate Phelps and Dorothy Mundy from the UTSW Imaging Core Facility and Dr. Patrina Pellett from GE Healthcare for their expertise with TIRF-SIM; Drs. Gregory Rak and Prachi Dongre for the generation of reporter plasmids and some of the NK92 cell lines; and Dr. David J. Williamson from King's College for sharing the color-blind safe lookup tables. This work was made possible in part through use of the Integrated Microscopy Core at Baylor College of Medicine, UT Southwest Live Cell Imaging Core Facility in Dallas, University of Manchester Flow Cytometry Core Facility, and Imaging Facility of the Texas Children's Hospital Center for Human Immunobiology. This work was supported by NIH-NIAID grant R01AI067946 (to J.S.O.). It was also supported by the following awards to D.M.D.: the Medical Research Council (award G1001044), a Wellcome Trust Investigator Award (110091), and the Manchester Collaborative Centre for Inflammation Research (funded by a pre-competitive open-innovation award from GSK, AstraZeneca, and The University of Manchester, UK).

AUTHOR CONTRIBUTIONS

A.F.C. and E.M.M. designed experiments, performed experiments, analyzed data, interpreted data, and wrote the manuscript. M.B.S. designed, performed, and analyzed experiments. J.S.O. and D.M.D. designed experiments and wrote the manuscript.

DECLARATION OF INTERESTS

The authors declare no competing interests. The LAMP1-pHluorin plasmid is patented as US20150212064/WO2013025598A1.

Received: August 14, 2017
Revised: November 13, 2017
Accepted: December 20, 2017
Published: February 1, 2018

REFERENCES

1. Mace, E.M., Dongre, P., Hsu, H.T., Sinha, P., James, A.M., Mann, S.S., Forbes, L.R., Watkin, L.B., and Orange, J.S. (2014). Cell biological steps and checkpoints in accessing NK cell cytotoxicity. Immunol. Cell Biol. *92*, 245–255.
2. Hsu, H.T., Mace, E.M., Carisey, A.F., Viswanath, D.I., Christakou, A.E., Wiklund, M., Önfelt, B., and Orange, J.S. (2016). NK cells converge lytic granules to promote cytotoxicity and prevent bystander killing. J. Cell Biol. *215*, 875–889.
3. Zhang, M., March, M.E., Lane, W.S., and Long, E.O. (2014). A signaling network stimulated by β2 integrin promotes the polarization of lytic granules in cytotoxic cells. Sci. Signal. *7*, ra96.
4. James, A.M., Hsu, H.T., Dongre, P., Uzel, G., Mace, E.M., Banerjee, P.P., and Orange, J.S. (2013). Rapid activation receptor- or IL-2-induced lytic granule convergence in human natural killer cells requires Src, but not downstream signaling. Blood *121*, 2627–2637.
5. Mentlik, A.N., Sanborn, K.B., Holzbaur, E.L., and Orange, J.S. (2010). Rapid lytic granule convergence to the MTOC in natural killer cells is dependent on dynein but not cytolytic commitment. Mol. Biol. Cell *21*, 2241–2256.
6. Valitutti, S., Dessing, M., Aktories, K., Gallati, H., and Lanzavecchia, A. (1995). Sustained signaling leading to T cell activation results from prolonged T cell receptor occupancy. Role of T cell actin cytoskeleton. J. Exp. Med. *181*, 577–584.
7. Varma, R., Campi, G., Yokosuka, T., Saito, T., and Dustin, M.L. (2006). T cell receptor-proximal signals are sustained in peripheral microclusters and terminated in the central supramolecular activation cluster. Immunity *25*, 117–127.
8. Orange, J.S., Ramesh, N., Remold-O'Donnell, E., Sasahara, Y., Koopman, L., Byrne, M., Bonilla, F.A., Rosen, F.S., Geha, R.S., and Strominger, J.L. (2002). Wiskott-Aldrich syndrome protein is required for NK cell cytotoxicity and colocalizes with actin to NK cell-activating immunologic synapses. Proc. Natl. Acad. Sci. USA *99*, 11351–11356.
9. Mace, E.M., Wu, W.W., Ho, T., Mann, S.S., Hsu, H.T., and Orange, J.S. (2012). NK cell lytic granules are highly motile at the immunological synapse and require F-actin for post-degranulation persistence. J. Immunol. *189*, 4870–4880.
10. Orange, J.S., Harris, K.E., Andzelm, M.M., Valter, M.M., Geha, R.S., and Strominger, J.L. (2003). The mature activating natural killer cell immunologic synapse is formed in distinct stages. Proc. Natl. Acad. Sci. USA *100*, 14151–14156.
11. Bouma, G., Mendoza-Naranjo, A., Blundell, M.P., de Falco, E., Parsley, K.L., Burns, S.O., and Thrasher, A.J. (2011). Cytoskeletal remodeling mediated by WASp in dendritic cells is necessary for normal immune synapse formation and T-cell priming. Blood *118*, 2492–2501.
12. Orange, J.S., Roy-Ghanta, S., Mace, E.M., Maru, S., Rak, G.D., Sanborn, K.B., Fasth, A., Saltzman, R., Paisley, A., Monaco-Shawver, L., et al. (2011). IL-2 induces a WAVE2-dependent pathway for actin reorganization that enables WASp-independent human NK cell function. J. Clin. Invest. *121*, 1535–1548.
13. Snapper, S.B., Rosen, F.S., Mizoguchi, E., Cohen, P., Khan, W., Liu, C.H., Hagemann, T.L., Kwan, S.P., Ferrini, R., Davidson, L., et al. (1998). Wiskott-Aldrich syndrome protein-deficient mice reveal a role for WASP in T but not B cell activation. Immunity *9*, 81–91.
14. Brown, A.C., Dobbie, I.M., Alakoskela, J.M., Davis, I., and Davis, D.M. (2012). Super-resolution imaging of remodeled synaptic actin reveals different synergies between NK cell receptors and integrins. Blood *120*, 3729–3740.
15. Brown, A.C., Oddos, S., Dobbie, I.M., Alakoskela, J.M., Parton, R.M., Eissmann, P., Neil, M.A., Dunsby, C., French, P.M., Davis, I., and Davis, D.M. (2011). Remodelling of cortical actin where lytic granules dock at natural killer cell immune synapses revealed by super-resolution microscopy. PLoS Biol. *9*, e1001152.
16. Rak, G.D., Mace, E.M., Banerjee, P.P., Svitkina, T., and Orange, J.S. (2011). Natural killer cell lytic granule secretion occurs through a pervasive actin network at the immune synapse. PLoS Biol. *9*, e1001151.
17. Abeyweera, T.P., Merino, E., and Huse, M. (2011). Inhibitory signaling blocks activating receptor clustering and induces cytoskeletal retraction in natural killer cells. J. Cell Biol. *192*, 675–690.
18. Culley, F.J., Johnson, M., Evans, J.H., Kumar, S., Crilly, R., Casasbuenas, J., Schnyder, T., Mehrabi, M., Deonarain, M.P., Ushakov, D.S., et al. (2009). Natural killer cell signal integration balances synapse symmetry and migration. PLoS Biol. *7*, e1000159.
19. Bunnell, S.C., Kapoor, V., Trible, R.P., Zhang, W., and Samelson, L.E. (2001). Dynamic actin polymerization drives T cell receptor-induced spreading: a role for the signal transduction adaptor LAT. Immunity *14*, 315–329.
20. Gomez, T.S., Kumar, K., Medeiros, R.B., Shimizu, Y., Leibson, P.J., and Billadeau, D.D. (2007). Formins regulate the actin-related protein 2/3 complex-independent polarization of the centrosome to the immunological synapse. Immunity *26*, 177–190.
21. Gomez, T.S., McCarney, S.D., Carrizosa, E., Labno, C.M., Comiskey, E.O., Nolz, J.C., Zhu, P., Freedman, B.D., Clark, M.R., Rawlings, D.J., et al. (2006). HS1 functions as an essential actin-regulatory adaptor protein at the immune synapse. Immunity *24*, 741–752.
22. Kaizuka, Y., Douglass, A.D., Varma, R., Dustin, M.L., and Vale, R.D. (2007). Mechanisms for segregating T cell receptor and adhesion molecules during immunological synapse formation in Jurkat T cells. Proc. Natl. Acad. Sci. USA *104*, 20296–20301.
23. Nolz, J.C., Gomez, T.S., Zhu, P., Li, S., Medeiros, R.B., Shimizu, Y., Burkhardt, J.K., Freedman, B.D., and Billadeau, D.D. (2006). The WAVE2 complex regulates actin cytoskeletal reorganization and CRAC-mediated calcium entry during T cell activation. Curr. Biol. *16*, 24–34.
24. Oddos, S., Dunsby, C., Purbhoo, M.A., Chauveau, A., Owen, D.M., Neil, M.A., Davis, D.M., and French, P.M. (2008). High-speed high-resolution imaging of intercellular immune synapses using optical tweezers. Biophys. J. *95*, L66–L68.
25. Babich, A., Li, S., O'Connor, R.S., Milone, M.C., Freedman, B.D., and Burkhardt, J.K. (2012). F-actin polymerization and retrograde flow drive sustained PLCγ1 signaling during T cell activation. J. Cell Biol. *197*, 775–787.
26. Campi, G., Varma, R., and Dustin, M.L. (2005). Actin and agonist MHC-peptide complex-dependent T cell receptor microclusters as scaffolds for signaling. J. Exp. Med. *202*, 1031–1036.
27. Kumari, S., Depoil, D., Martinelli, R., Judokusumo, E., Carmona, G., Gertler, F.B., Kam, L.C., Carman, C.V., Burkhardt, J.K., Irvine, D.J., and Dustin, M.L. (2015). Actin foci facilitate activation of the phospholipase C-γ in primary T lymphocytes via the WASP pathway. eLife *4*, e04953.
28. Yi, J., Wu, X.S., Crites, T., and Hammer, J.A., III. (2012). Actin retrograde flow and actomyosin II arc contraction drive receptor cluster dynamics at the immunological synapse in Jurkat T cells. Mol. Biol. Cell *23*, 834–852.
29. Ashdown, G.W., Burn, G.L., Williamson, D.J., Pandžić, E., Peters, R., Holden, M., Ewers, H., Shao, L., Wiseman, P.W., and Owen, D.M. (2017). Live-cell super-resolution reveals F-actin and plasma membrane dynamics at the T cell synapse. Biophys. J. *112*, 1703–1713.
30. Fritzsche, M., Fernandes, R.A., Chang, V.T., Colin-York, H., Clausen, M.P., Felce, J.H., Galiani, S., Erlenkämper, C., Santos, A.M., Heddleston, J.M., et al. (2017). Cytoskeletal actin dynamics shape a ramifying actin network underpinning immunological synapse formation. Sci. Adv. *3*, e1603032.

31. Murugesan, S., Hong, J., Yi, J., Li, D., Beach, J.R., Shao, L., Meinhardt, J., Madison, G., Wu, X., Betzig, E., and Hammer, J.A. (2016). Formin-generated actomyosin arcs propel T cell receptor microcluster movement at the immune synapse. J. Cell Biol. *215*, 383–399.

32. Tran, D.T., Masedunskas, A., Weigert, R., and Ten Hagen, K.G. (2015). Arp2/3-mediated F-actin formation controls regulated exocytosis in vivo. Nat. Commun. *6*, 10098.

33. Stray-Pedersen, A., Jouanguy, E., Crequer, A., Bertuch, A.A., Brown, B.S., Jhangiani, S.N., Muzny, D.M., Gambin, T., Sorte, H., Sasa, G., et al. (2014). Compound heterozygous CORO1A mutations in siblings with a mucocutaneous-immunodeficiency syndrome of epidermodysplasia verruciformis-HPV, molluscum contagiosum and granulomatous tuberculoid leprosy. J. Clin. Immunol. *34*, 871–890.

34. Mace, E.M., and Orange, J.S. (2014). Lytic immune synapse function requires filamentous actin deconstruction by Coronin 1A. Proc. Natl. Acad. Sci. USA *111*, 6708–6713.

35. Alter, G., Malenfant, J.M., and Altfeld, M. (2004). CD107a as a functional marker for the identification of natural killer cell activity. J. Immunol. Methods *294*, 15–22.

36. Betts, M.R., Brenchley, J.M., Price, D.A., De Rosa, S.C., Douek, D.C., Roederer, M., and Koup, R.A. (2003). Sensitive and viable identification of antigen-specific CD8+ T cells by a flow cytometric assay for degranulation. J. Immunol. Methods *281*, 65–78.

37. Grakoui, A., Bromley, S.K., Sumen, C., Davis, M.M., Shaw, A.S., Allen, P.M., and Dustin, M.L. (1999). The immunological synapse: a molecular machine controlling T cell activation. Science *285*, 221–227.

38. Monks, C.R., Freiberg, B.A., Kupfer, H., Sciaky, N., and Kupfer, A. (1998). Three-dimensional segregation of supramolecular activation clusters in T cells. Nature *395*, 82–86.

39. Stinchcombe, J.C., Majorovits, E., Bossi, G., Fuller, S., and Griffiths, G.M. (2006). Centrosome polarization delivers secretory granules to the immunological synapse. Nature *443*, 462–465.

40. Gustafsson, N., Culley, S., Ashdown, G., Owen, D.M., Pereira, P.M., and Henriques, R. (2016). Fast live-cell conventional fluorophore nanoscopy with ImageJ through super-resolution radial fluctuations. Nat. Commun. *7*, 12471.

41. Hetrick, B., Han, M.S., Helgeson, L.A., and Nolen, B.J. (2013). Small molecules CK-666 and CK-869 inhibit actin-related protein 2/3 complex by blocking an activating conformational change. Chem. Biol. *20*, 701–712.

42. Nolen, B.J., Tomasevic, N., Russell, A., Pierce, D.W., Jia, Z., McCormick, C.D., Hartman, J., Sakowicz, R., and Pollard, T.D. (2009). Characterization of two classes of small molecule inhibitors of Arp2/3 complex. Nature *460*, 1031–1034.

43. Rizvi, S.A., Neidt, E.M., Cui, J., Feiger, Z., Skau, C.T., Gardel, M.L., Kozmin, S.A., and Kovar, D.R. (2009). Identification and characterization of a small molecule inhibitor of formin-mediated actin assembly. Chem. Biol. *16*, 1158–1168.

44. Butler, B., and Cooper, J.A. (2009). Distinct roles for the actin nucleators Arp2/3 and hDia1 during NK-mediated cytotoxicity. Curr. Biol. *19*, 1886–1896.

45. Andzelm, M.M., Chen, X., Krzewski, K., Orange, J.S., and Strominger, J.L. (2007). Myosin IIA is required for cytolytic granule exocytosis in human NK cells. J. Exp. Med. *204*, 2285–2291.

46. Sanborn, K.B., Mace, E.M., Rak, G.D., Difeo, A., Martignetti, J.A., Pecci, A., Bussel, J.B., Favier, R., and Orange, J.S. (2011). Phosphorylation of the myosin IIA tailpiece regulates single myosin IIA molecule association with lytic granules to promote NK-cell cytotoxicity. Blood *118*, 5862–5871.

47. Sanborn, K.B., Rak, G.D., Maru, S.Y., Demers, K., Difeo, A., Martignetti, J.A., Betts, M.R., Favier, R., Banerjee, P.P., and Orange, J.S. (2009). Myosin IIA associates with NK cell lytic granules to enable their interaction with F-actin and function at the immunological synapse. J. Immunol. *182*, 6969–6984.

48. Lagrue, K., Carisey, A., Oszmiana, A., Kennedy, P.R., Williamson, D.J., Cartwright, A., Barthen, C., and Davis, D.M. (2013). The central role of the cytoskeleton in mechanisms and functions of the NK cell immune synapse. Immunol. Rev. *256*, 203–221.

49. Moulding, D.A., Record, J., Malinova, D., and Thrasher, A.J. (2013). Actin cytoskeletal defects in immunodeficiency. Immunol. Rev. *256*, 282–299.

50. Mace, E.M., and Orange, J.S. (2012). Dual channel STED nanoscopy of lytic granules on actin filaments in natural killer cells. Commun. Integr. Biol. *5*, 184–186.

51. Ritter, A.T., Asano, Y., Stinchcombe, J.C., Dieckmann, N.M., Chen, B.C., Gawden-Bone, C., van Engelenburg, S., Legant, W., Gao, L., Davidson, M.W., et al. (2015). Actin depletion initiates events leading to granule secretion at the immunological synapse. Immunity *42*, 864–876.

52. Fritzsche, M., Li, D., Colin-York, H., Chang, V.T., Moeendarbary, E., Felce, J.H., Sezgin, E., Charras, G., Betzig, E., and Eggeling, C. (2017). Self-organizing actin patterns shape membrane architecture but not cell mechanics. Nat. Commun. *8*, 14347.

53. Mendoza, M.C., Besson, S., and Danuser, G. (2012). Quantitative fluorescent speckle microscopy (QFSM) to measure actin dynamics. Curr. Protoc. Cytom. *62*, 2.18.1–2.18.26.

54. Wulfing, C., Purtic, B., Klem, J., and Schatzle, J.D. (2003). Stepwise cytoskeletal polarization as a series of checkpoints in innate but not adaptive cytolytic killing. Proc. Natl. Acad. Sci. USA *100*, 7767–7772.

55. Sinai, P., Nguyen, C., Schatzle, J.D., and Wülfing, C. (2010). Transience in polarization of cytolytic effectors is required for efficient killing and controlled by Cdc42. Proc. Natl. Acad. Sci. USA *107*, 11912–11917.

56. Beal, A.M., Anikeeva, N., Varma, R., Cameron, T.O., Vasiliver-Shamis, G., Norris, P.J., Dustin, M.L., and Sykulev, Y. (2009). Kinetics of early T cell receptor signaling regulate the pathway of lytic granule delivery to the secretory domain. Immunity *31*, 632–642.

57. Jenkins, M.R., Tsun, A., Stinchcombe, J.C., and Griffiths, G.M. (2009). The strength of T cell receptor signal controls the polarization of cytotoxic machinery to the immunological synapse. Immunity *31*, 621–631.

58. Basu, R., Whitlock, B.M., Husson, J., Le Floc'h, A., Jin, W., Oyler-Yaniv, A., Dotiwala, F., Giannone, G., Hivroz, C., Biais, N., et al. (2016). Cytotoxic T cells use mechanical force to potentiate target cell killing. Cell *165*, 100–110.

59. Ritter, A.T., Kapnick, S.M., Murugesan, S., Schwartzberg, P.L., Griffiths, G.M., and Lippincott-Schwartz, J. (2017). Cortical actin recovery at the immunological synapse leads to termination of lytic granule secretion in cytotoxic T lymphocytes. Proc. Natl. Acad. Sci. USA *114*, E6585–E6594.

60. Schindelin, J., Arganda-Carreras, I., Frise, E., Kaynig, V., Longair, M., Pietzsch, T., Preibisch, S., Rueden, C., Saalfeld, S., Schmid, B., et al. (2012). Fiji: an open-source platform for biological-image analysis. Nat. Methods *9*, 676–682.

61. Dormann, D., Libotte, T., Weijer, C.J., and Bretschneider, T. (2002). Simultaneous quantification of cell motility and protein-membrane-association using active contours. Cell Motil. Cytoskeleton *52*, 221–230.

62. Mangeol, P., Prevo, B., and Peterman, E.J. (2016). KymographClear and KymographDirect: two tools for the automated quantitative analysis of molecular and cellular dynamics using kymographs. Mol. Biol. Cell *27*, 1948–1957.

63. Motulsky, H.J., and Brown, R.E. (2006). Detecting outliers when fitting data with nonlinear regression—a new method based on robust nonlinear regression and the false discovery rate. BMC Bioinformatics *7*, 123.

STAR★METHODS

KEY RESOURCES TABLE

REAGENT or RESOURCE	SOURCE	IDENTIFIER
Antibodies		
CD8-Bv785 (staining concentration 6 µg/mL)	Biolegend	Cat#301045; Clone RPTA-T8; Lots B187390, B221662
CD45RA-Bv421 (staining concentration 6 µg/mL)	Biolegend	Cat#304129; Clone HT100; Lot B204474
CD45RO-PE (staining concentration 5 µg/mL)	Biolegend	Cat#304205; Clone UCHL1; Lot B183218
CD107a-AF647 (staining concentration 1.25 µg/mL)	Biolegend	Cat#328612; Clone H4A3; Lot E11642-1632
Perforin-AF488 (staining concentration 20 µg/mL)	Biolegend	Cat#308108; Clone dG9; Lot B198112
CD3 (LEAF) (concentration 5 µg/mL)	Biolegend	Cat#317315; Clone OKT3; Lot B211929
CD28 (concentration 5 µg/mL)	Biolegend	Cat#302914; Clone CD28.2; Lot B219506
CD18 (concentration 5 µg/mL)	Hybridoma	Clone TS1/18; Single batch
CD18 (concentration 5 µg/mL)	Hybridoma	Clone IB4; Single batch
NKp30 (LEAF, CD337) (concentration 5 µg/mL)	Biolegend	Cat#325204; Clone P30-15; Lot B224416
NKp30 (CD337) (staining concentration 5 µg/mL)	R&D Systems Europe	Cat#MAB18491; Clone 210847; Lot JRK0216081
Chemicals, Peptides, and Recombinant Proteins		
rh ICAM-1 (concentration 5 µg/mL)	R&D Systems	Cat#ADP4-200; Lot WV1915121
Phalloidin AF488 (staining concentration 3U/mL)	Thermo Fisher	Cat#A12379; Lot 1816955
Phalloidin AF532 (staining concentration 3U/mL)	Thermo Fisher	Cat#A22282; Lot 1417648
LysoTracker Red DND-99 (staining concentration 1/1000)	Thermo Fisher	Cat#L7528; Lot 983858
Cell Proliferation Dye eFluor 670 (staining concentration 1/1000)	eBioscience	Cat#65-0840-85; Lot 4297564
SYTOX Orange Nucleic Acid Stain (staining concentration 0.2 µM)	Thermo Fisher	Cat# S11368; Lot 1488607
DMSO, Anhydrous (dilution 1/500, 1/1000)	Thermo Fisher	Cat#D12345
Blebbistatin (concentration 40 µM)	Sigma Aldrich	Cat#B0560-1MG; Lot SLBM5499V
CK666 (concentration 50 µM)	Calbiochem	Cat#182515-25MG; Lot 264765
Jasplakinolide (concentration 1 µM)	ChemCruz	Cat#sc-202191A; Lot A0417
SMIFH2 (concentration 50 µM)	Sigma Aldrich	Cat#S4826-5MG; Lot 075M4601V
Critical Commercial Assays		
Human CD8+ T cell enrichment kit	Stemcell	Cat#15063; Lot 16F72180
T Cell Expansion Kit, human	Miltenyi Biotec	Cat#130-091-441; Lot 5150227115
NK Cell Isolation Kit, human	Miltenyi Biotec	Cat#130-092-657; Lot 5170608527
LookOut mycoplasma PCR detection kit	Sigma Aldrich	Cat#MP0035-1KT
Amaxa Kit R	Lonza	Cat#VCA-1001
Experimental Models: Cell Lines		
NK92	ATCC	Cat#CRL-2407
Experimental Models: Organisms/Strains		
Human: healthy donor	Sample size is indicated in the legend of each relevant figure.	All samples were acquired with approval from the Institutional Review Boards of Texas Children's Hospital and University of Manchester under the guidelines of the Declaration of Helsinki.
Recombinant DNA		
LAMP1-pHluorin plasmid	[16]	N/A
mApple-LAMP1-pHluorin-N-8 plasmid	Davidson Collection (unpublished)	AddGene Plasmid #54918

(*Continued on next page*)

Continued

REAGENT or RESOURCE	SOURCE	IDENTIFIER
LifeAct plasmid	Dr Janis Burkhardt (University of Pennsylvania)	N/A
LeGO-E Emerald-GFP plasmid	Dr. Boris Fehse	AddGene Plasmid #27359
mTurquoise plasmid	Dr. Theodorus Gadella (University of Amsterdam)	N/A
Software and Algorithms		
Imaris (v8.4.1 and v9.0.2)	Bitplane	http://www.bitplane.com/
MATLAB (v2016b and v2017b)	The MathWorks	https://www.mathworks.com/
Huygens (v16.10)	Scientific Volume Imaging	https://svi.nl/HomePage
Volocity (v6.3)	Perkin Elmer	http://cellularimaging.perkinelmer.com/downloads/detail.php?id=14
Fiji v1.51n	[60]	https://fiji.sc/
NanoJ toolbox for ImageJ	[40]	https://bitbucket.org/rhenriqueslab/nanoj-core/wiki/Home
QuimP toolbox for ImageJ v17.04.04	[61]	https://www2.warwick.ac.uk/fac/sci/dcs/people/till_bretschneider/quimp/
KymographClear Macro toolset for ImageJ	[62]	https://sites.google.com/site/kymographanalysis/
LookUp Tables	Dr David J Williamson (King's College, London)	https://github.com/quokka79/DavLUT
Actin Mesh Analyzer	[14, 15] and this paper	https://github.com/alexcarisey/ActinMeshAnalyzer
GraphPad Prism 7.03	GraphPad Software	https://www.graphpad.com/
DSS Research online tool (statistical power calculation)	DSS Research	https://www.dssresearch.com/KnowledgeCenter/toolkitcalculators/statisticalpowercalculators.aspx
Illustrator CC 22.0.1 (64-bit)	Adobe Systems Incorporated	http://www.adobe.com/products/illustrator.html

CONTACT FOR REAGENT AND RESOURCE SHARING

Further information and requests for resources and reagents should be directed to and will be fulfilled by the Lead Contact, Jordan S. Orange, MD PhD (orange@bcm.edu). The LAMP1-pHluorin plasmid is patented as US20150212064/WO2013025598A1.

EXPERIMENTAL MODEL AND SUBJECT DETAILS

Cell lines and cell culture

NK92 cell line was obtained from the ATCC and was maintained in alpha minimum modified Eagle medium, 0.2 mM myoinositol, 0.1 mM beta-mercaptoethanol, 0.02 mM folic acid, 12.5% heat inactivated horse serum, 12.5% heat-inactivated FBS (Sigma Aldrich), 2 mM L-glutamine and non-essential amino acids (ThermoFisher Scientific), supplemented with 100 U/mL Il-2 (Roche). NK92 expressing LAMP1-pHluorin cells were generated as described previously [16] and maintained as above. HeLa cells were cultivated in Dulbecco's modified Eagle's medium with high glucose, supplemented with 10% of heat-inactivated FBS (Sigma Aldrich), 2 mM L-glutamine and non-essential amino acids (ThermoFisher Scientific). All cell lines were maintained in 37°C, 5% CO2 tissue culture incubators and routinely confirmed to be mycoplasma negative using LookOut mycoplasma PCR detection kit (Sigma Aldrich). Functional validation of the LAMP1-pHluorin cell line was performed by flow cytometry to ensure detection of fluorescence following NK cell degranulation as previously described [16].

Primary cell isolation

T cells were isolated from peripheral blood of adult healthy donors using Pan T cell isolation kit (Miltenyi Biotec). To generate CTL subsets, T cells were sorted (BD Influx). Live, single, $CD8^+$ cells (clone RPA-T8) were two-way sorted into $CD45RO^+$ (clone UCHL1) or $CD45RA^+$ (clone HI100). Cells were rested overnight at 37°C in clone media: Dulbecco's modified Eagle's medium supplemented with 10% human serum, 2 mM L-glutamine, 1 mM sodium pyruvate, 1 mM penicillin/streptomycin and 1 mM non-essential amino acids (all obtained from ThermoFisher Scientific). NK cells were isolated from peripheral blood using NK cell negative isolation kit (Miltenyi Biotec). For experiments using fresh NK cells, cells were maintained in clone media at 37°C and used 2-4 hr after isolation. Rested NK cells were maintained in clone media supplemented with 150 units/mL recombinant human IL-2 (Roche) and

were used 5-6 days after isolation. All samples were acquired with approval from the Institutional Review Boards of Texas Children's Hospital and University of Manchester under the guidelines of the Declaration of Helsinki.

METHOD DETAILS

Plasmids and transfection

mApple-LAMP1-pHluorin-N-8 was a gift from Dr. Michael Davidson (Addgene #54918). A LifeAct expressing plasmid was a gift from Dr. Janis Burkhardt (University of Pennsylvania). The LeGO-E plasmid containing Emerald-GFP was a gift from Dr. Boris Fehse (Addgene #27359) and LifeAct was cloned into BamHI and EcoRI restriction sites to create LifeAct.mEmerald. mTurquoise was a gift from Dr. Theodorus Gadella (University of Amsterdam). LifeAct.mTurquoise was generated by cloning LifeAct into the XhoI and EcoRI restriction sites of MIGR1 mTurquoise. NK92 cell lines were generated by retroviral transduction as previously described [16] or nucleofection using Amaxa Kit R per manufacturer's instructions (Lonza). Positive cells were amplified under antibiotic selection pressure and were sorted for low, intermediate or high expression of the fluorescently tagged protein on an Aria II Fluorescence Activated Cell Sorter (BD). Each sorted population was then used for pilot experiments to determine the lowest possible expression level required for optimal imaging conditions by confocal, STED, TIRF or SIM.

Sample preparation for microscopy

NK or T cells were activated on #1.5 coverslips (Corning) or LabTek imaging chambers (Nunc) pre-coated with either 5 μg/mL anti-CD18 (clones IB4, TS1/18), anti-NKp30 (clones P30-15, 210847), recombinant human ICAM-1 (R&D Systems), anti-CD3 (clone OKT3) or anti-CD28 (clone CD28.2). Cell activation was performed at 37°C in pre-warmed media. Following activation, cells were fixed using BD CytoFix/CytoPerm or 4% PFA with 0.1% Triton X-100 at room temperature then gently washed with PBS 1% BSA and 0.1% Saponin buffer (Sigma Aldrich). Staining for F-actin was performed in this buffer using phalloidin AlexaFluor 488, or phalloidin AlexaFluor 532 with anti-perforin antibody directly conjugated to AlexaFluor 488 (clone dG9). Coverslips were mounted using ProLong Gold antifade reagent (ThermoFisher Scientific) and slides were cured for 18-24 hr prior to imaging for STED microscopy. For SIM microscopy, Vectashield H-1000 (Vector Laboratories) was used instead. For primary T and NK cell experiments and for live imaging, mounting media was not used and slides were imaged immediately after preparation.

All live imaging experiments were performed after washing the cells twice and replacing the growth medium with phenol red free RPMI-1640 medium, supplemented 20mM HEPES (Sigma Aldrich), 2mM L-glutamine, non-essential amino acids (ThermoFisher Scientific) adjusted to pH 7.3. Before use, the medium was supplemented with 100 U/mL Il-2 (Roche) if needed. When indicated, cells were loaded with 1/1000 of LysoTracker Red DND-99 (ThermoFisher Scientific) for 30 min at 37°C before being washed three times or a directly conjugated monoclonal antibody anti-CD107a (clone H4A3) was added to the imaging medium. HeLa cells were labeled with eFluor670 to allow their detection in the live conjugation experiments. In all experiments involving cytoskeletal inhibitors, cells were seeded and incubated for 15 min prior to the addition of an equivalent volume of pre-warmed medium containing blebbistatin, CK666, jasplakinolide or DMSO (all from Sigma Aldrich) as a vehicle control at double the final concentration (final concentrations: 40 μM blebbistatin, 50 μM CK666, 1 μM jasplakinolide, 50 μM SMIFH2). On all systems, live imaging was performed at 37°C using environmental chambers or stage top inserts.

Confocal and STED microscopy

Images were acquired through an HCX PL APO 100 × /1.40 NA oil objective on a Leica TCS SP8 STED 3X laser scanning confocal microscope (Leica Microsystems). Excitation was performed by sequential combination using pulsed white-light laser and emission was detected using time-gated HyD detectors operating in standard mode. When indicated, STED depletion lasers (592 nm or 660 nm) were applied to obtain higher resolution for the dyes AlexaFluor 488 and AlexaFluor 532 (STED 592 nm) or for the fluorescent proteins mTurquoise and mEmerald (STED 660nm). Images were acquired by LASAF software v3.3 and exported for processing and analysis as raw data. Fixed cell images were deconvolved using CMLE algorithm in Huygens (v16.10, Scientific Volume Imaging) with a signal-to-noise ratio of 10.

TIRF microscopy live imaging acquisition

Images were acquired through an APO N TIRF 60 × /1.49 NA oil objective on an Olympus IX81. Excitation by 488 nm (Spectra Physics) and 561 nm (Cobolt) lasers was combined using an LMM5 laser merge module and delivered to a Spectral Diskovery TIRF (Oxford Instruments) with an identical penetration depth set to 150 nm for all wavelengths. Image acquisition by a C9100 EM-CCD camera (Hamamatsu) was handled by MetaMorph (v7.8.3).

Cell viability assay following drug treatment

NK92 cells were activated on #1 LabTek imaging chambers (Nunc) pre-coated with 5 μg/mL anti-CD18 (clone IB4) and anti-NKp30 (clones P30-15, 210847). Cell activation was performed at 37°C in pre-warmed imaging media for 15 min prior to the addition of an equivalent volume of pre-warmed imaging medium containing blebbistatin, CK666, jasplakinolide, SMIFH2, DMSO as a vehicle control, and Triton X-100 as a positive control (all from Sigma Aldrich) at double the final concentration (final concentrations: 40 μM blebbistatin, 50 μM CK666, 1 μM jasplakinolide, 50 μM SMIFH2, 1/500 DMSO, 0.2% Triton X-100). After 10 min of incubation in presence of the drug, the cells were washed twice with pre-warmed imaging medium before being covered with pre-warmed

imaging medium containing 0.2 μM SYTOX orange (ThermoFisher Scientific). After 5 min of incubation, a 3x3 tile scan (1.72 × 1.72 mm^2) was acquired by confocal microscopy through an HC PL APO CS2 20 × /0.75 NA immersion objective on a Leica TCS SP8 STED 3X laser scanning confocal microscope. Excitation was performed using pulsed white-light laser and emission was detected using time-gated HyD detectors operating in standard mode, alongside with the transmitted light channel. Images were acquired by LASAF software v3.3 and exported for processing and analysis in Fiji. Images were analyzed using a custom script. Briefly, binary masks of individual cells were created using the threshold tool after applying a bandpass filter (10-30 pixels range) to the transmitted light channel. Cell outlines were filtered to remove incorrectly identified debris and shadows using size and circularity cut-offs (25-400μm^2 and 0.6-1.0, respectively). Intensity of SYTOX channel was then measured for each cell using the masks and plotted using GraphPad Prism.

Fixed SIM and live TIRF-SIM microscopy

Fixed cells were imaged using a GE DeltaVision OMX v3 with Blaze SIM module in SIM illumination mode. Fluorescence was collected through a 60 × /1.4 NA oil objective and captured on a sCMOS camera at 95 MHz across a 512 × 512 pixels area with no binning and a camera pixel size of 80 nm under the control of SoftWorx 6.5.2. Each frame was reconstructed using 3 orientations and 5 phase shifts and a Wiener filter constant of 0.005 before applying a Gaussian filter with a sigma value of 80 nm. The final reconstructed image has a pixel size of 40nm. Live cells were imaged using a GE OMX SR microscope in TIRF-SIM illumination mode. Fluorescence was collected through a 60 × /1.4 NA oil objective and captured on a sCMOS camera at 286 MHz across a 1024 × 1024 pixels area with no binning and a camera pixel size of 80 nm under the control of SoftWorx 6.5.2. Each frame was reconstructed using 3 orientations and 3 phase shifts and a Wiener filter constant of 0.005 before applying a Gaussian filter with a sigma value of 80 nm. The final reconstructed image has a pixel size of 40 nm.

Parameters of live cell imaging experiments

Figure	Method	Frame rate	Duration	Pixel size (final image)	Average FWHM measured using a thin fibrillar structure
1A	Confocal	Every 15 s	30 min	89 nm	-
1B and 1C	Confocal	Every 51-80 s	60 min	113 nm	-
1D	Confocal	Every 15 s	30 min	89 nm	-
1E	Confocal	Every 89 s	90 min	180 nm xy, 800 nm z	-
1F	TIRF-SIM	Every 10 s	10 min	40 nm	130 nm
1G	TIRF	1 frame	-	65 nm	250 nm
2A	STED	Fixed	-	40 nm	120 nm
2B	STED	Fixed	-	40 nm	120 nm
2C	SIM	Fixed	-	40 nm	130 nm
2D	STED	Every 10 s	3 min	30 nm	150 nm
2E	STED	Fixed	-	30 nm	120 nm
3	TIRF-SIM	Every 5 s	5 min	40 nm	130 nm
4A–4C	TIRF-SIM	Every 5 s	5 min	40 nm	130 nm
4D	STED	Fixed	-	30 nm	120 nm
4E	TIRF-SIM	Every 5 s	Discontinuous (drug treatment, washout)	40 nm	130 nm
5A–5C	STED	Fixed	-	30 nm	150 nm
5D	TIRF	Every 1 s	5 min	65 nm	250 nm
5E and 5F	STED	Every 10 s	3 min	50 nm	180 nm
5G–5I	STED	Fixed	-	30 nm	150 nm
6	STED	Every 10 s	3 min	30 nm	150 nm
S1A	Confocal	Every 51-80 s	60 min	113 nm	-
S1B	Confocal	Every 2min	60 min	113 nm	-
S1C	3D-SIM	Fixed	-	40 nm xy, 125 nm z	130 nm xy, 300 nm z
S1D	3D-STED	Fixed	-	30 nm xy, 100 nm z	120 nm xy, 250 nm z
S2	STED	Fixed	-	30 nm	120 nm
S3A and S3B	TIRF-SIM	Every 3 s	1 min	40 nm	-
S3C and S3D	STED	Every 10 s	2 min	50 nm	-
S3E	TIRF-SIM	Every 5 s	5 min	40 nm	130 nm

(*Continued on next page*)

Continued

Figure	Method	Frame rate	Duration	Pixel size (final image)	Average FWHM measured using a thin fibrillar structure
S3F	STED	Every 10 s	2 min	50 nm	180 nm
S3G	STED	Every 10 s	2 min	50 nm	180 nm
S3H	STED	Every 1 s	15 s	50 nm	180 nm
S3I	STED	Every 10 s	1 min 50 s	50 nm	180 nm
S3J	TIRF-SIM	Every 5 s	5 min	40 nm	130 nm
S3K	TIRF-SIM	Every 5 s	5 min	40 nm	130 nm
S4A	Confocal	1 frame	-	568 nm	-
S4B	TIRF-SIM	Every 5 s	Discontinuous for drug treatment and washout	40 nm	130 nm
S4C–S4E	Confocal	Every 1 min	Discontinuous for drug treatment and washout	101 nm	230 nm
S5	TIRF	Every 1 s	5 min	65 nm	250 nm

Image analysis

Line profile

Brightness and contrast in live cell images were uniformly thresholded prior to analysis using Fiji (v1.51n). Line profiles were generated in Fiji and measurements were exported to Prism for visualization.

Measurement of actin clearances

Detection and measurement of F-actin clearances was performed as described previously [16]. In summary, images were imported to Fiji and background subtracted using Rolling Ball Subtraction with a radius of 50 pixels. Pixel intensities were squared twice. An ROI (50-100 μm^2) was drawn in the central region of the synapse and thresholded using the default threshold in Fiji with "dark background" unchecked. Clearances were detected using the Analyze Particles function, with a clearance with an area of 0.05 μm^2 considered permissive for a 150 nm diameter lytic granule with the assumption of uniform circularity. Clearance measurements (number, area) were exported to Excel and the frequency of clearances was measured per μm^2 based upon the area of the ROI for the given cell. For live cell imaging series, this algorithm was applied to each frame individually. Multichannel images were split prior to analysis of only the F-actin channel. Measurement of mean hole area and granule penetrable area were made from STED, SIM and TIRF-SIM datasets using an updated version of the MATLAB app previously published [15]. The Orientation Filter Transform (OFT) was part of the NanoJ toolbox for ImageJ [40].

Quantification of LifeAct fluorescent signal

Measurement of LifeAct fluorescence signal intensity and NK92 cell footprint over time was performed by thresholding the raw data using the fluorescence channel information and using the Analyze Particles / Measurement functions in Fiji. When indicated, the footprint of the cell over time was normalized using the lowest value measured and the maximum area reached by each cell when the spreading measurement reaches a plateau. Measurement of the spreading speed of the NK92 cells onto the different coated surfaces was done by extracting the coefficient from the linear fit of the spreading area curve. The portion of the curve used for the fit is limited by the first time point when the cell is visible until the first time point when the cell has reached its maximum spreading area (plateau). All values were analyzed and plotted in Prism. Normalized sizes using 0% as the smallest footprint detected and 100% as the maximum footprint size are presented in Figure S1A.

Measurement of the edge of the IS

Segmentation and plotting of the intensity of the LifeAct fluorescence signal in the 2 μm wide outer rim of the cell was obtained using QuimP plugin [61] for Fiji (v17.04.04).

Kymograph analysis

Kymographs analyzing the fluorescent signal over time were prepared using the Reslice tool in Fiji or using the KymographClear toolbox for Fiji [62] when extraction of the stable, forward and backward components was necessary.

Optical flow analysis

Raw images obtained by TIRF-SIM were subjected to linear signal scaling before being exported as 8-bit TIFF stacks using Fiji. A custom MATLAB script using Farnback algorithm for optical flow (opticalFlowFarneback function, implemented since r2015b) was then applied (NumPyramidLevels = 3, PyramidScale = 0.5, NumIteration = 3, NeighborhoodSize = 5, FilterSize = 15) and the resulting flow object was plotted with a decimation factor of 5 and a scaling value of 2.

Population-based degranulation measurement

Detection of lytic granules and degranulation of NK92.LAMP1-pHluorin for Figure 1G was performed using image segmentation and detection algorithms in Volocity (PerkinElmer). The frequency of degranulation was calculated as the number of cells with a positive

LAMP1-pHluorin signal over the total number of cells observed within 75 min of imaging following addition of cells to imaging chambers. Events smaller than 0.05 μm^2 were excluded from analysis and fluorescent thresholds were uniformly applied to each cell within a given experiment.

Degranulation analysis

NK92.mApple-LAMP1-pHluorin cells were plated on activating surface for 20 min and treated with cytoskeletal inhibitors for 10 min. In the following 15 min, 3 time lapses of 5 min each were acquired (1 frame/sec) and the number of degranulation in each cell was measured as follows. Raw multichannel acquired by TIRF microscopy were imported into Imaris (v8.4.1, Bitplane). All vesicles present in the mApple channel were segmented and tracked using the "Spot" tool with the following settings: size 0.5 μm, local background subtraction, auto quality setting filtering and default settings for tracking (1 frame gap allowed). The mean intensity of the pHluorin signal within each object was measured throughout the lifetime of the trajectory and the standard deviation of the intensity over time was calculated. Each object displaying a standard deviation equal to more than two times the standard deviation of a non-degranulating object was considered as a degranulation event after manual confirmation by visual inspection.

Figure preparation

Acquired images from microscopy based experiments were subjected to signal re-scaling using linear transformation using Fiji [60] for display in the figures. All figures were prepared using Illustrator CC 22.0.1 (Adobe Systems).

QUANTIFICATION AND STATISTICAL ANALYSIS

Sample sizes were determined using statistical power calculator with an alpha error level of 5% (DSS Research online tool). From the initiation of the study, datasets were tested for outliers using robust regression and outlier removal (ROUT) with a Q value of 1% [63]. Data was assessed for normality using D'Agostino and Pearson omnibus normality test and if criteria for Gaussian distribution were not met, Mann-Whitney tests were applied to compare datasets with two samples (two-tailed). Ordinary one-way ANOVA test for multiple comparisons with Tukey's post hoc test was used for multiple comparisons of groups with normal distribution. Unpaired two-tailed Student's t test was used for comparison of two samples with normal distribution. Welch's correction was applied in case of unequal variance. A *p* value of less than 0.05 was considered significant. Statistical analyses and graphing were performed using Prism (v7.03, GraphPad). All boxplots graphs presented in this study indicate the mean value and the standard deviation for the population considered.

Figure	Sample size and repeat	Statistical test	Outlier removal
1A	Representative images from 3 independent repeats with 10 cells each time.	N/A	N/A
1B	N = 37, 21 and 35 cells respectively per condition from 3, 4 and 4 independent experiments respectively	One-way ANOVA Kruskal-Wallis test (Dunn's)	No
1C	N = 37, 21 and 35 cells respectively per condition from 3, 4 and 4 independent experiments respectively	Ordinary one-way ANOVA with Tukey's post hoc comparison	No
1D	Representative cells from Figure 1A	N/A	N/A
1E	Representative images selected from 3 independent repeats	N/A	N/A
1F	Representative images selected from 43 cells from 6 experiments	N/A	N/A
1G	N = 60, 67, 64 respectively from 3 independent experiments	N/A	N/A
2A	Representative images selected from more than 65 cells per condition pooled from 5 independent repeats	N/A	N/A
2B	N = 58, 59, 59 and 59 cells respectively per condition pooled from 4 independent repeats	Ordinary one-way ANOVA with Tukey's post hoc comparison	No
2C	N = 20 cells per condition from 1 experiment representative of 3 independent repeats	One-way ANOVA Kruskal-Wallis test (Dunn's)	ROUT 1%
2D	Representative images selected from 20 cells from 2 independent repeats	N/A	N/A
2E	N = 10, 13, 24, 37, 41, 50, 49, 50 and 61 cells respectively pooled from 3 independent repeats	One-way ANOVA Kruskal-Wallis test (Dunn's)	No
3A	Representative images selected from 43 cells from 6 independent repeats	N/A	N/A
3B	Representative images selected from 43 cells from 6 independent repeats	N/A	N/A

(*Continued on next page*)

Continued

Figure	Sample size and repeat	Statistical test	Outlier removal
3C	Quantification from dataset presented in Figures 3B and 3D	N/A	N/A
3D	Representative images selected from 43 cells from 6 independent repeats	N/A	N/A
3E	Quantification from dataset presented in Figures 3B and 3D	N/A	N/A
3F	Quantification from dataset presented in Figures 3B and 3D	N/A	N/A
4A	Representative images selected from 43 cells from 6 independent repeats	N/A	N/A
4B	Details from dataset presented in Figure 4A	N/A	N/A
4C	N = 20 cells from 4 independent repeats	N/A	No
4D	N = 10 and 13 cells respectively from 1 experiment representative of 4 independent repeats	Mann-Whitney, two-tailed test	No
4E	Representative images selected from 12 cells from 4 independent repeats	N/A	N/A
5A	Representative images selected from more than 100 cells imaged from 4 independent repeats	N/A	N/A
5B	N = 41 per condition from 2 pooled independent experiments representative of 4 independent repeats	Unpaired t-test with Welch's correction, two-tailed	ROUT 1%
5C	N = 50 per condition from 2 pooled independent experiments representative of 4 independent repeats	Unpaired Mann-Whitney, two-tailed test	ROUT 1%
5D	N = 41, 28, 31 and 15 cells respectively per condition pooled from 3 independent repeats	One-way ANOVA Kruskal-Wallis test (Dunn's)	ROUT 1%
5E	Representative images selected from 24 cells from 4 experiments	N/A	N/A
5F	Representative images selected from 24 cells from 4 experiments	N/A	N/A
5G	Representative images selected from 41 and 38 cells respectively from 4 experiments	N/A	N/A
5H	N = 41 and 38 cells respectively from two pooled independent experiments representative of 4 repeats	Unpaired Mann-Whitney, two-tailed test	ROUT 1%
5I	N = 48 and 39 cells respectively from two pooled independent experiments representative of 4 repeats	Unpaired Mann-Whitney, two-tailed test	ROUT 1%
6A	Data representative of 24 cells from 3 independent experiments.	N/A	N/A
6B	Data representative of 24 cells from 3 independent experiments.	N/A	N/A
6C	Data representative of 24 cells from 3 independent experiments (MFI of actin at the location of 6 granules normalized to their arrival, 1 example of granule in red)	N/A	N/A
6D	Data representative of 24 cells from 3 independent experiments (MFI of actin at the location of 1 granule normalized to their arrival, 1 example of granule in red)	N/A	N/A
S1A	N = 37, 21 and 35 cells respectively per condition from 3, 4 and 4 independent experiments respectively	N/A	N/A
S1B	Representative images selected from 7 independent repeats	N/A	N/A
S1C	Representative image selected from 1 repeat from 2C	N/A	N/A
S1D	Representative image selected from 1 repeat from 2A	N/A	N/A
S2A	Representative images selected from 17 cells	N/A	N/A
S2B	N = 11 cells pooled from 2 independent repeats	N/A	N/A
S2C	N = 11 cells pooled from 2 independent repeats	N/A	N/A
S2D	Representative images selected from S2EF	N/A	N/A
S2E	N = 10 cells per condition per donor for 4 donors, each donor colored individually	One-way ANOVA Kruskal-Wallis test (Dunn's)	No

(*Continued on next page*)

Continued

Figure	Sample size and repeat	Statistical test	Outlier removal
S2F	N = 10 cells per condition per donor for 4 donors, each donor colored individually	One-way ANOVA Kruskal-Wallis test (Dunn's)	No
S2G	Representative images selected from S2HI	N/A	N/A
S2H	N = 10 cells per condition per donor for 4 donors, each donor colored individually	One-way ANOVA Kruskal-Wallis test (Dunn's)	No
S2I	N = 10 cells per condition per donor for 4 donors, each donor colored individually	One-way ANOVA Kruskal-Wallis test (Dunn's)	No
S2J	Representative images selected from S2KL	N/A	N/A
S2K	N = 10 cells per condition per donor for 3 donors, each donor colored individually	One-way ANOVA Kruskal-Wallis test (Dunn's)	No
S2L	N = 10 cells per condition per donor for 3 donors, each donor colored individually	One-way ANOVA Kruskal-Wallis test (Dunn's)	No
S2M	Representative images selected from S2NO	N/A	N/A
S2N	N = 10 cells per condition per donor for 4 donors, each donor colored individually	One-way ANOVA Kruskal-Wallis test (Dunn's)	No
S2O	N = 10 cells per condition per donor for 4 donors, each donor colored individually	One-way ANOVA Kruskal-Wallis test (Dunn's)	No
S3A	N = 33 cells pooled from 6 independent repeats	N/A	N/A
S3B	N = 33 cells pooled from 6 independent repeats	N/A	N/A
S3C	N = 6 cells from 1 experiment representative of 3 independent repeats	N/A	N/A
S3D	N = 7 cells from 1 experiment representative of 3 independent repeats	Unpaired Mann-Whitney, two-tailed test	No
S3E	Representative images selected from 43 cells from 6 independent repeats	N/A	N/A
S3F	Representative images selected from 25 cells from 3 independent experiments	N/A	N/A
S3G	N = 5 cells from 1 experiment representative of 3 independent repeats	N/A	N/A
S3H	N = 6 cells from 1 experiment representative of 3 independent repeats	N/A	N/A
S3I	N = 10 cells from 1 experiment representative of 3 independent repeats	Unpaired Mann-Whitney, two-tailed test	No
S3J	Representative images selected from 43 cells from 6 independent repeats	N/A	N/A
S3K	Representative images selected from 28 cells from 3 experiments	N/A	N/A
S4A	N = 3846, 3451, 2358, 3829, 5124, 2476 and 4125 cells respectively from a representative experiment of 2 repeats.	N/A	N/A
S4B	Representative images selected from 12 cells from 4 independent repeats	N/A	N/A
S4C	N = 10 cells from a representative experiment out of 6 independent repeats (error bars represent 95% CI).	N/A	N/A
S4D	N = 10 cells from a representative experiment out of 6 independent repeats (error bars represent 95% CI).	N/A	N/A
S4E	N = 10 cells from a representative experiment out of 6 independent repeats (error bars represent 95% CI).	N/A	N/A
S5A	N/A	N/A	N/A
S5B	N = 6 representative traces from 11 cells from dataset used in Figures S5D–S5I (DMSO)	N/A	N/A
S5C	Representative images selected from 11 cells from dataset used in Figures S5D–S5I (DMSO)	N/A	N/A

(*Continued on next page*)

Continued

Figure	Sample size and repeat	Statistical test	Outlier removal
S5D	N = 11 and 15 cells respectively from 1 experiment representative of 3 independent repeats	Unpaired t-test with Welch's correction, two-tailed	No
S5E	N = 11 and 15 cells respectively from 1 experiment representative of 3 independent repeats	Unpaired t-test with Welch's correction, two-tailed	No
S5F	N = 11 and 15 cells respectively from 1 experiment representative of 3 independent repeats	N/A	No
S5G	N = 11 and 15 cells respectively from 1 experiment representative of 3 independent repeats	N/A	No
S5H	N = 11 and 15 cells respectively from 1 experiment representative of 3 independent repeats	N/A	No
S5I	N = 11 and 15 cells respectively from 1 experiment representative of 3 independent repeats	N/A	No

Current Biology, Volume *28*

Supplemental Information

Nanoscale Dynamism of Actin Enables Secretory Function in Cytolytic Cells

Alexandre F. Carisey, Emily M. Mace, Mezida B. Saeed, Daniel M. Davis, and Jordan S. Orange

Figure S1

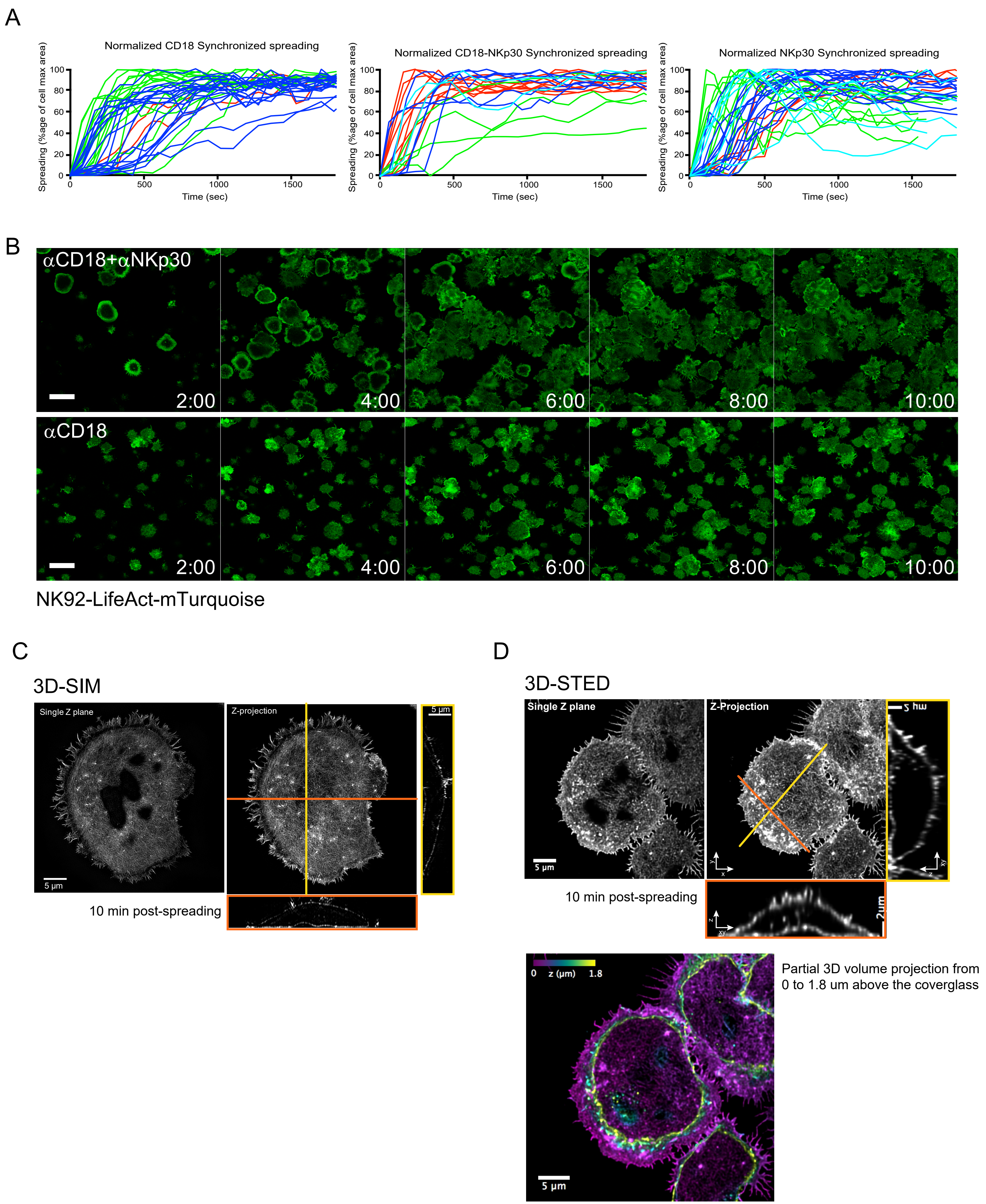
A
Normalized CD18 Synchronized spreading
Normalized CD18-NKp30 Synchronized spreading
Normalized NKp30 Synchronized spreading
Spreading (%age of cell max area)
Time (sec)
0
20
40
60
80
100
500
1000
1500
B
αCD18+αNKp30
αCD18
2:00
4:00
6:00
8:00
10:00
NK92-LifeAct-mTurquoise
C
3D-SIM
Single Z plane
Z-projection
5 µm
10 min post-spreading
D
3D-STED
Single Z plane
Z-Projection
5 µm
2µm
10 min post-spreading
0
z (µm)
1.8
Partial 3D volume projection from
0 to 1.8 um above the coverglass

Figure S1. Mature NK cell lytic synapse is defined by a pervasive actin network. Related to Figure 1.

(A) Individual spreading measurements traces for NK92.LifeAct-mEmerald cells plated on the indicated substrate and imaged by confocal microscopy at the plane of the glass. Each track has been aligned in time to the beginning of each cell contact with the glass and normalized to the maximum cell area achieved. N = 37, 21 and 35 cells respectively per condition from 3, 4 and 4 independent experiments. **(B)** Representative frames (from 7 independent repeats) from time lapse imaging of larger fields of view of NK92.LifeAct-mEmerald cells plated on the indicated substrate, imaged by confocal microscopy at the plane of the glass showing the consistency of the detection of a bright circular lamellipodium in presence of anti-NKp30 antibody. Scale bar = 20 µm. **(C)** 3D-SIM reconstruction of NK92 cell stained for F-actin by phalloidin 488 and its orthogonal projection illustrating the irregularity of the plasma membrane of activated NK cells. Scale bar = 5 µm. **(D)** Similar sample as **(C)** imaged by 3D-STED microscopy. Scale bar = 5 µm.

Figure S2

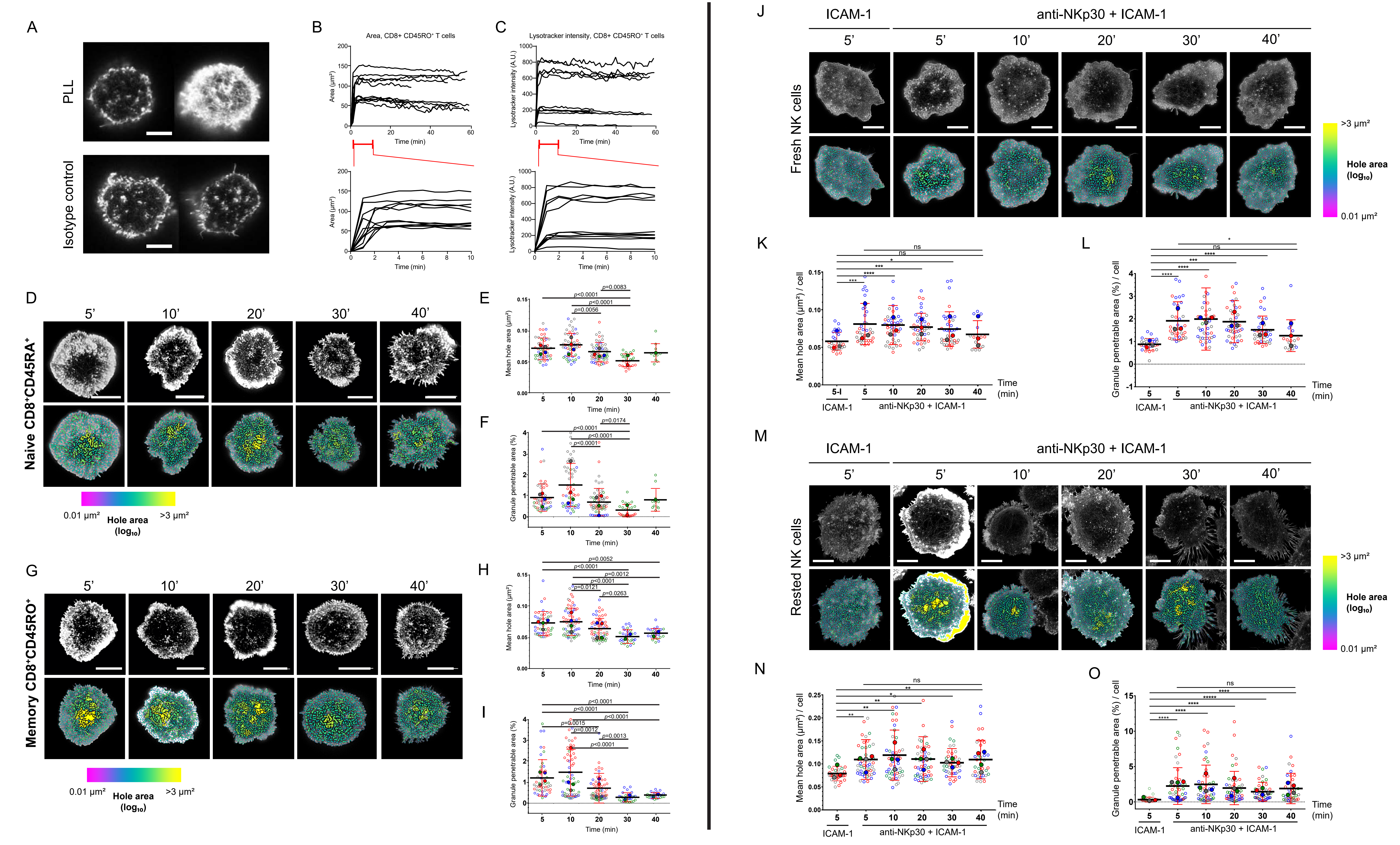
A
PLL
Isotype control
B
Area, CD8+ CD45RO+ T cells
Area (µm²)
Time (min)
C
Lysotracker intensity, CD8+ CD45RO+ T cells
Lysotracker intensity (A.U.)
Time (min)
D
5'
10'
20'
30'
40'
Naive CD8+CD45RA+
0.01 µm²
Hole area (log₁₀)
>3 µm²
E
Mean hole area (µm²)
p<0.0001
p=0.0083
p<0.0001
p=0.0056
Time (min)
F
Granule penetrable area (%)
p<0.0001
p=0.0174
p<0.0001
p<0.0001
Time (min)
G
Memory CD8+CD45RO+
0.01 µm²
Hole area (log₁₀)
>3 µm²
H
Mean hole area (µm²)
p=0.0052
p<0.0001
p=0.0012
p<0.0001
p=0.0121
p=0.0263
Time (min)
I
Granule penetrable area (%)
p<0.0001
p<0.0001
p<0.0001
p=0.0015
p=0.0012
p=0.0013
p<0.0001
Time (min)
J
ICAM-1
anti-NKp30 + ICAM-1
Fresh NK cells
>3 µm²
Hole area (log₁₀)
0.01 µm²
K
Mean hole area (µm²) / cell
5-I
ICAM-1
anti-NKp30 + ICAM-1
Time (min)
L
Granule penetrable area (%) / cell
ICAM-1
anti-NKp30 + ICAM-1
Time (min)
M
ICAM-1
anti-NKp30 + ICAM-1
Rested NK cells
>3 µm²
Hole area (log₁₀)
0.01 µm²
N
Mean hole area (µm²) / cell
ICAM-1
anti-NKp30 + ICAM-1
Time (min)
O
Granule penetrable area (%) / cell
ICAM-1
anti-NKp30 + ICAM-1
Time (min)

Figure S2. Granule-permissive sized clearances persist following degranulation and are a feature of primary cytotoxic T cells and NK cells. Related to Figure 2.

(A) Representative images (selected from 17 cells) of primary T cells plated on PLL or isotype control coated glass for 20 minutes and stained for F-actin, illustrating the dense cortical actin mesh observed in the absence of activation signal. Scale bar = 5 µm. **(B)** Individual spreading measurements traces for primary $CD8^+$ $CD45RO^+$ cells loaded with LysoTracker Red and plated on anti-CD3 and anti-CD28 and imaged by confocal microscopy at the plane of the glass. Each track has been aligned in time to the beginning of each cell contact with the glass. Below, an inset shows the first 10 minutes of the time lapse. **(C)** Measurement of LysoTracker fluorescence intensity at the plane of the synapse during the time lapse imaging experiment described in **(B)**. N = 11 cells pooled from 2 independent repeats in (B) and **(C)**. Primary human naïve [$CD8^+$ $CD45RA^+$] **(D)** or memory [$CD8^+$ $CD45RO^+$] **(G)** T cells isolated from peripheral blood of healthy donors and activated on anti-CD3 and anti-CD28 for times indicated prior to fixation and staining with Phalloidin AlexaFluor 488. Cells were imaged by time-gated STED microscopy. Scale bar = 5 µm. Mean hole area per cell was plotted on the images (lower panel, heat map) and is shown in graphs on right **(E, H)** along the percentage of the granule penetrable area per cell **(F, I)**. Images shown are representative of those from 4 healthy donors which were pooled for quantitative analysis. N = 10 cells per donor, each donor colored individually in graphs. *p* value calculated using one-way ANOVA Kruskal-Wallis test (Dunn's). Primary human fresh **(J)** or rested **(M)** NK cells isolated from peripheral blood of healthy donors and activated on ICAM-1 or a combination of ICAM-1 and anti-NKp30 for times indicated prior to fixation and staining with Phalloidin AlexaFluor 488. Cells were imaged by time-gated STED microscopy. Scale bar = 5 µm. Mean hole area per cell was plotted back on the images (lower panel, heat map) and is shown in graphs directly below **(K, N)** along the percentage of the granule penetrable area per cell **(L, O)**. Images shown are representative of those from 3 (fresh NK cells) or 4 (rested NK cells) healthy donors which were pooled for quantitative

analysis. N = 10 cells per donor, each donor is colored individually in graphs. *p* value calculated using one-way ANOVA Kruskal-Wallis test (Dunn's).

Figure S3

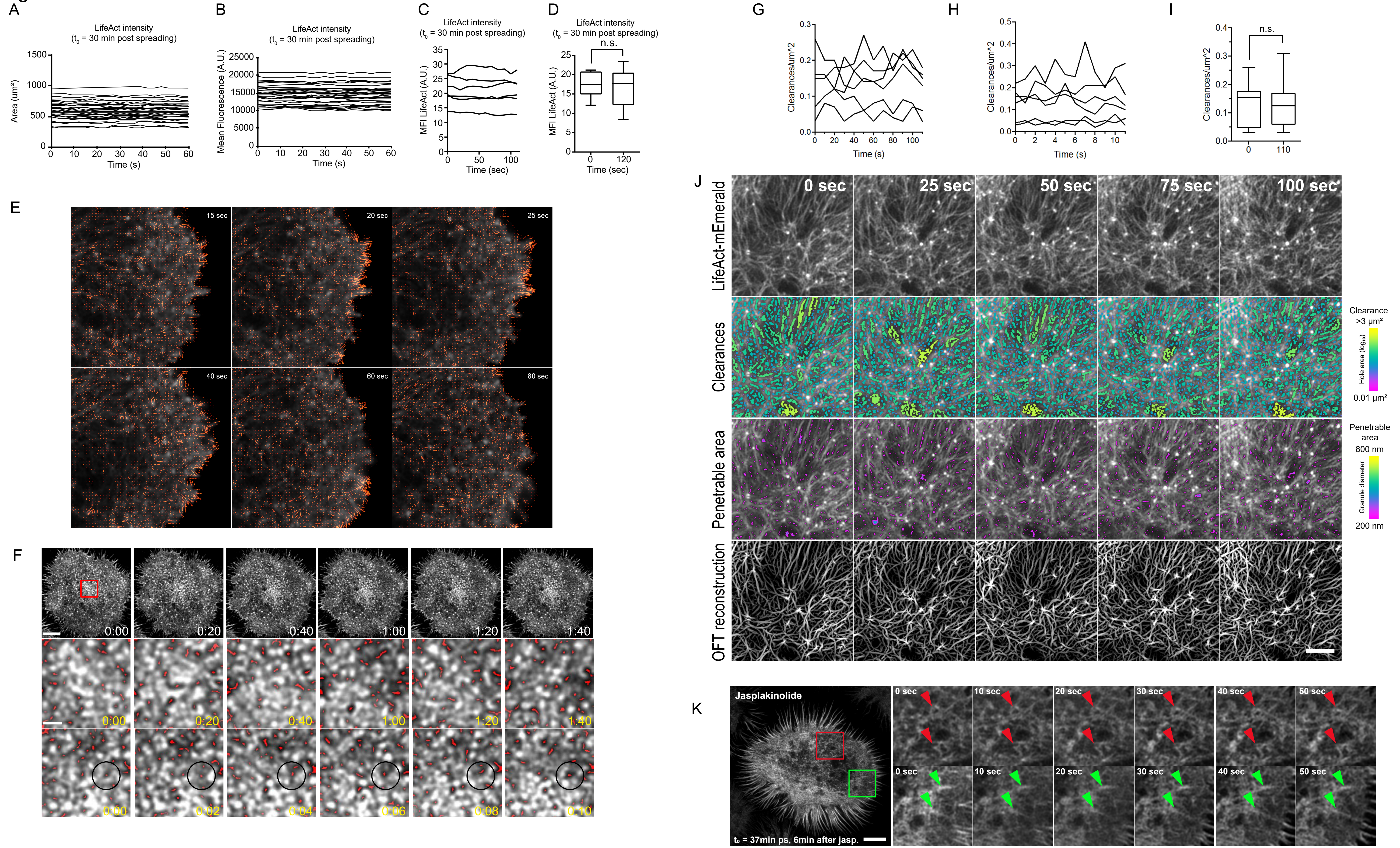

A
LifeAct intensity
(t_0 = 30 min post spreading)
Area (um²)
Time (s)
B
LifeAct intensity
(t_0 = 30 min post spreading)
Mean Fluorescence (A.U.)
Time (s)
C
LifeAct intensity
(t_0 = 30 min post spreading)
MFI LifeAct (A.U.)
Time (sec)
D
LifeAct intensity
(t_0 = 30 min post spreading)
n.s.
MFI LifeAct (A.U.)
Time (sec)
E
15 sec
20 sec
25 sec
40 sec
60 sec
80 sec
F
0:00
0:20
0:40
1:00
1:20
1:40
0:02
0:04
0:06
0:08
0:10
G
Clearances/um^2
Time (s)
H
Clearances/um^2
Time (s)
I
n.s.
Clearances/um^2
J
LifeAct-mEmerald
Clearances
Penetrable area
OFT reconstruction
0 sec
25 sec
50 sec
75 sec
100 sec
Clearance
>3 µm²
Hole area (log₁₀)
0.01 µm²
Penetrable area
800 nm
Granule diameter
200 nm
K
Jasplakinolide
t₀ = 37min ps, 6min after jasp.
0 sec
10 sec
20 sec
30 sec
40 sec
50 sec

Figure S3. Global F-actin stability at the mature NK IS is coupled to local actin dynamism. Related to Figure 3.

NK92 LifeAct.mTurquoise cells were activated for 30 minutes prior to initiation of imaging by TIRF-SIM or time gated STED and reached a stable footprint size **(A)**. Filamentous actin was detected using LifeAct-mEmerald and mean fluorescence intensity over time throughout the time of imaging was measured in TIRF-SIM **(B)** or time gated STED **(C)**. **(D)** The MFI of LifeAct did not change significantly over the time course of imaging as calculated from 7 cells from 1 experiment (representative of 3 experiments). *p* value calculated by Mann-Whitney unpaired two-tailed test. **(E)** Frames from the optical flow analysis of the LifeAct-mEmerald fluorescent signal at the edge of NK92 cell following 30 minutes of activation on antibody-coated glass. Optical flow is calculated using Farnback's algorithm for every frame (5 seconds) but displayed at critical timepoints illustrating the concomitance of actin flow with membrane edge movement (see Movie S3). **(F)** Time lapse series of a single cell representative of 25 cells from 3 independent experiments imaged by live time-gated STED microscopy. Insets below are from the same cell but captured at different frame rate (1 frame every 10 seconds or 1 frame per second) to illustrate the fast and continuous dynamism. Scale bar = 5 µm (top panel), 0.5 µm (bottom panel). **(G, H)** Measurement of the clearances/μm^2 over time confirms the dynamism of the clearances observed in **(F)** using both slow (1 frame every 10 seconds, 5 cells, **G**) and fast datasets (1 frame every second, 6 cells, **H**). **(I)** The overall distribution of the number of clearances did not change significantly over the time course of imaging (110 seconds, 1 frame per second) as calculated from 10 cells from 1 experiment (representative of 3 experiments). *p* value calculated by Mann-Whitney unpaired two-tailed test. **(J)** Larger field of view from the dataset presented in Figure 3D and in Movie S4. Below the fluorescent LifeAct-mEmerald reporter signal, the clearances have been plotted onto the original dataset. The third row shows the clearances compatible with the extrusion of a granule with a diameter ranging from 200 to 800nm. The last row displays the OFT reconstruction of the same area emphasizing the local dynamism of the actin fibers. Scale bar = 1 µm. **(K)** Stabilization of the F-actin

cytoskeleton with 1 µM jasplakinolide administered 30 minutes after activation (see movie S5). Arrowheads highlight stable F-actin structures. Representative cell from 28 cells from 3 independent repeats.

Figure S4

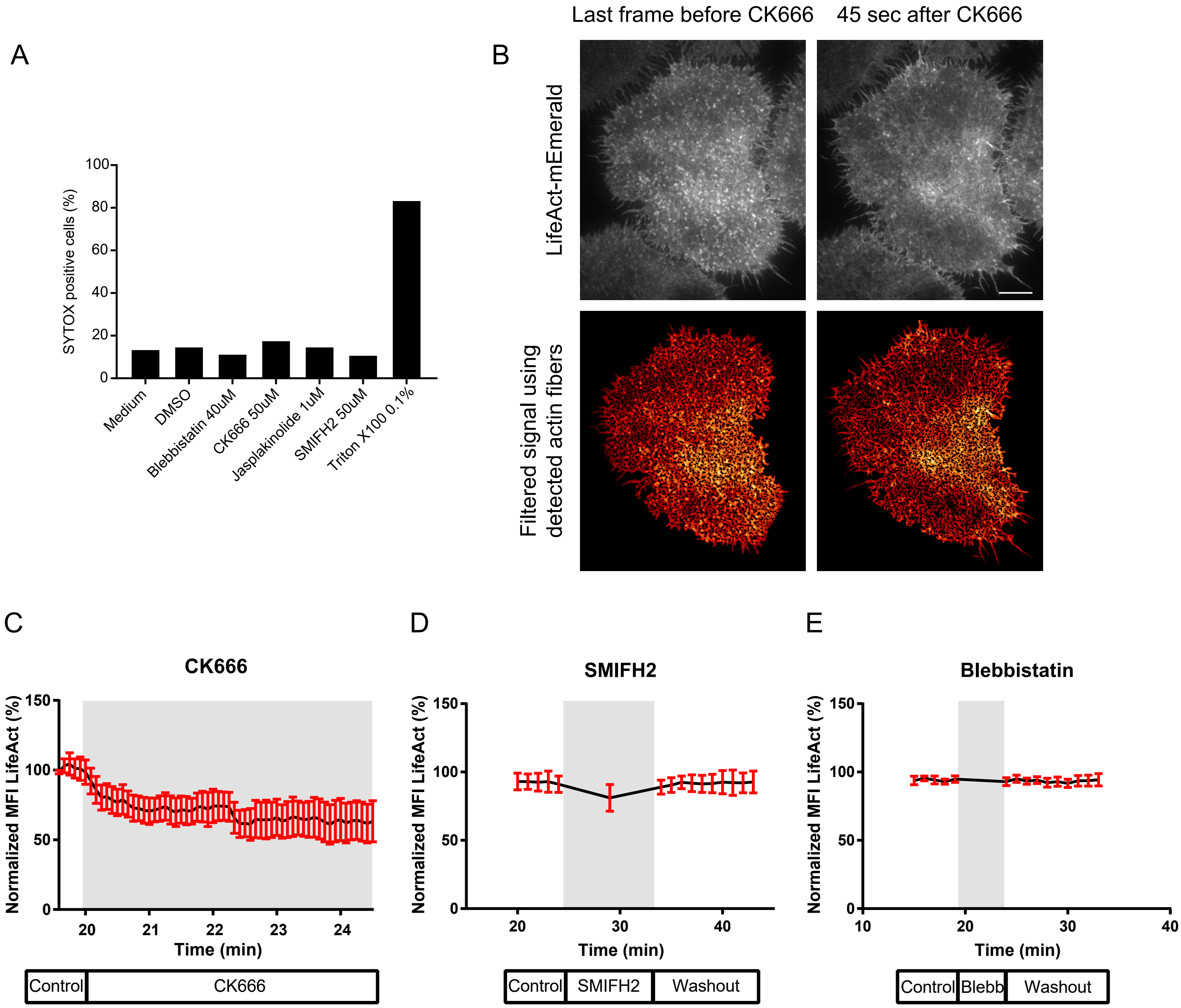
A
SYTOX positive cells (%)
100
80
60
40
20
0
Medium
DMSO
Blebbistatin 40uM
CK666 50uM
Jasplakinolide 1uM
SMIFH2 50uM
Triton X100 0.1%
B
Last frame before CK666
45 sec after CK666
LifeAct-mEmerald
Filtered signal using detected actin fibers
C
CK666
Normalized MFI LifeAct (%)
150
100
50
0
20
21
22
23
24
Time (min)
Control
CK666
D
SMIFH2
Normalized MFI LifeAct (%)
150
100
50
0
20
30
40
Time (min)
Control
SMIFH2
Washout
E
Blebbistatin
Normalized MFI LifeAct (%)
150
100
50
0
10
20
30
40
Time (min)
Control
Blebb
Washout

Figure S4. The mature immune synapse is characterized by intense Arp2/3 based actin remodeling. Related to Figure 4.

(A) Viability of NK92.LifeAct-mEmerald cells tested after treatment with the indicating drug for 10 minutes following 20 minutes of activation on antibody coated glass. SYTOX was added while washing out the drug and imaged by confocal microscopy. The proportion of SYTOX positive cells indicates cell death (Triton X100 is a positive control). None of the drug treatment used in this study is significantly more toxic to NK92 cells than the control (medium) or vehicle control (DMSO). **(B)** Detection of the actin filaments from the TIRF-SIM dataset used in Figure 4E illustrating the increased sized of the clearances and disappearance of the actin foci following the addition of 50 µM of CK666 to activated NK92 cells on antibody coated glass (see Movie S7). Scale bar = 5 µm. **(C)** Normalized mean fluorescence intensity of the LifeAct-mEmerald signal at the immune synapse of NK92 cells activated for 20 minutes on antibody coated glass and treated with 50 µM of CK666. Cells are imaged every 5 seconds and the intensity is normalized to 100% for each cell 5 seconds before the addition of the inhibitor. Data from 10 cells, representative from 6 repeats. Error bars indicate 95% CI. **(D)** Normalized mean fluorescence intensity of the LifeAct-mEmerald signal at the immune synapse of NK92 cells activated for 20 minutes on antibody coated glass and treated with 50 µM of SMIFH2. Cells are imaged every minute and the intensity is normalized to 100% for each cell 5 minutes before the addition of the inhibitor. One time point is recorded during the treatment and then for 10 minutes, every minute, after drug washout. No significant change in the total actin mass at the IS is observed during or after the drug treatment. Data from 10 cells, representative from 6 repeats. Error bars indicate 95% CI. **(E)** Normalized mean fluorescence intensity of the LifeAct-mEmerald signal at the immune synapse of NK92 cells activated for 20 minutes on antibody coated glass and treated with 40 µM of blebbistatin. Cells are imaged every minute and the intensity is normalized to 100% for each cell 5 minutes before the addition of the inhibitor. Then, cells are imaged every minute for 10 minutes after drug washout. No

significant change in the total actin mass at the IS is observed after the drug treatment. Data from 10 cells, representative from 6 repeats. Error bars indicate 95% CI.

Figure S5

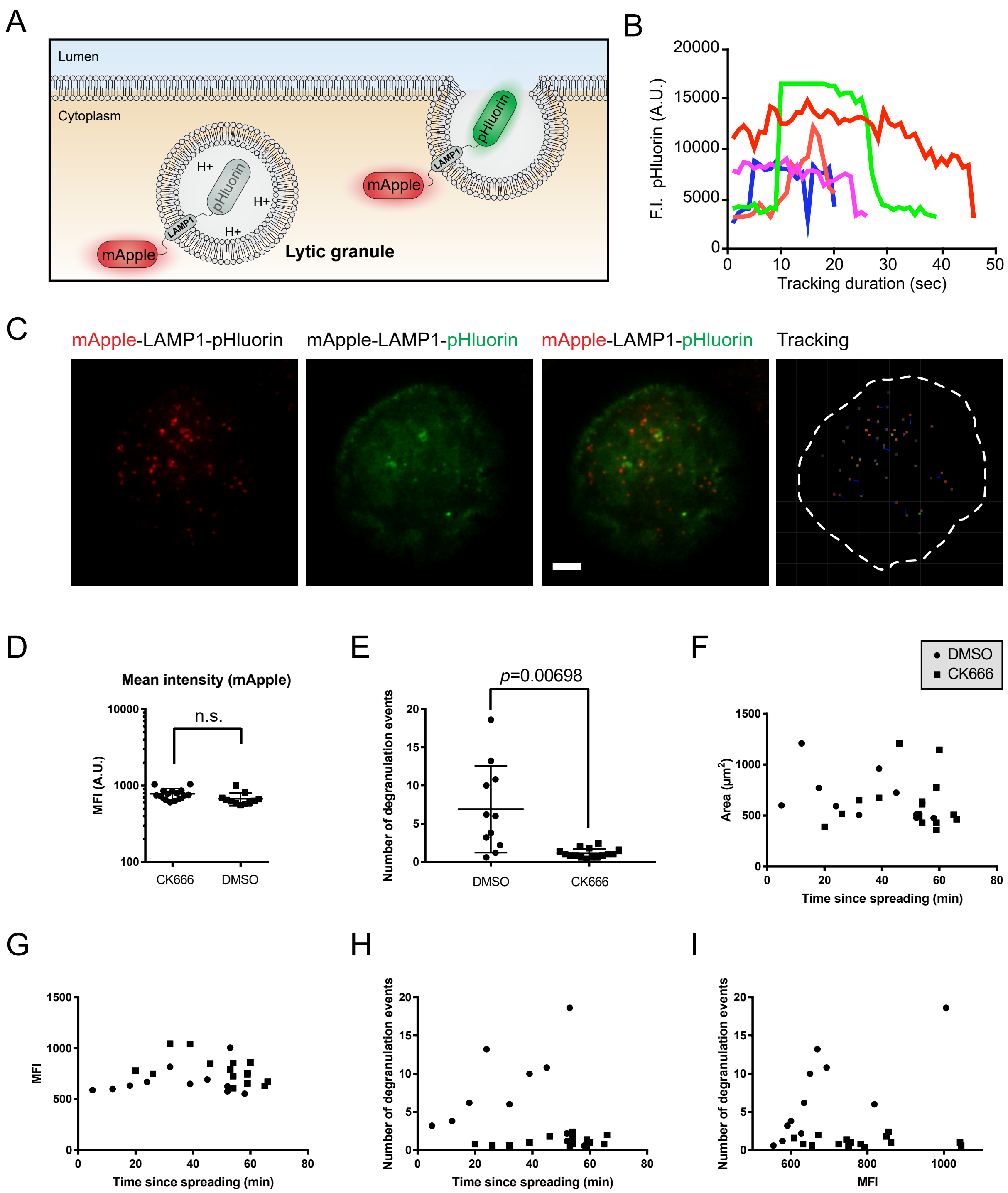
A
Lumen
Cytoplasm
H+
pHluorin
H+
H+
LAMP1
mApple
Lytic granule
pHluorin
LAMP1
mApple
B
F.I. pHluorin (A.U.)
Tracking duration (sec)
C
mApple-LAMP1-pHluorin
mApple-LAMP1-pHluorin
mApple-LAMP1-pHluorin
Tracking
D
Mean intensity (mApple)
n.s.
MFI (A.U.)
CK666
DMSO
E
p=0.00698
Number of degranulation events
DMSO
CK666
F
DMSO
CK666
Area (μm²)
Time since spreading (min)
G
MFI
Time since spreading (min)
H
Number of degranulation events
Time since spreading (min)
I
Number of degranulation events
MFI

Figure S5. Analysis of degranulation using mApple-LAMP1-pHluorin expressing NK92. Related to Figure 5.

(A) Cartoon illustrating the mode of action of the mApple-LAMP1-pHluorin degranulation sensor. **(B)** Example of 6 representative individual traces of the intensity of the pHluorin signal within individual vesicles segmented using the mApple fluorescent signal. True degranulation events are confirmed by a sudden surge in pHluorin signal when the pH inside the vesicle is neutralized after fusion with the lumen at the plasma membrane. **(C)** Dual color TIRF time lapse of NK92.mApple-LAMP1-pHluorin expressing cell activated on anti-CD18 and anti-NKp30 antibodies coated glass. The two fluorescent signals are presented separately before the merged panel and the segmented vesicles. Scale bar = 2 µm. **(D)** An entire dataset of NK92.mApple-LAMP1-pHluorin expressing cell activated on anti-CD18 and anti-NKp30 antibodies coated glass has been analyzed to demonstrate the absence of bias between expression level of the degranulation sensor and the probability of observation of degranulation events (N = 11 and 15 cells for DMSO and CK666 treated cells, respectively). Cells were sorted for an intermediate level of expression of the degranulation sensor using the mApple intensity. **(E)** The treatment with CK666 confirmed the same results as obtained in the main figure of the manuscript (Figure 5D). *p* value calculated by unpaired t-test with Welch's correction two-tailed test. No correlation was observed between the time of the acquisition of the 5 minutes long time lapse for degranulation count and the area of the cell **(F)** or the MFI of the sensor itself **(G)**, confirming that our time window is limited to fully spread NK cells with a mature IS. **(H)** Interestingly, we could reproduce the results of Figure 1G and observe an increase of the number of degranulation events over time in the control (DMSO treated) condition only. **(I)** Finally, the number of degranulation event observed was not correlated with the level of the expression of the sensor.
